# A Review of Molecular Imaging of Glutamate Receptors

**DOI:** 10.3390/molecules25204749

**Published:** 2020-10-16

**Authors:** Jong-Hoon Kim, János Marton, Simon Mensah Ametamey, Paul Cumming

**Affiliations:** 1 Neuroscience Research Institute, Gachon University, Incheon 21565, Korea; 2 Gachon Advanced Institute for Health Science and Technology, Graduate School, Incheon 21565, Korea; 3 Department of Psychiatry, Gil Medical Center, Gachon University College of Medicine, Gachon University, Incheon 21565, Korea; 4 ABX Advanced Biochemical Compounds, Biomedizinische Forschungsreagenzien GmbH, Heinrich-Glaeser-Strasse 10-14, D-1454 Radeberg, Germany; marton@abx.de; 5 Centre for Radiopharmaceutical Sciences ETH-PSI-USZ, Institute of Pharmaceutical Sciences ETH, Vladimir-Prelog-Weg 4, CH-8093 Zürich, Switzerland; simon.ametamey@pharma.ethz.ch; 6 Department of Nuclear Medicine, University of Bern, Inselspital, Freiburgstrasse 18, CH-3010 Bern, Switzerland; 7 School of Psychology and Counselling, Queensland University of Technology, Brisbane QLD 4059, Australia

**Keywords:** glutamate receptors, positron emission tomography, single photon emission computed tomography, radioligands

## Abstract

Molecular imaging with positron emission tomography (PET) and single photon emission computed tomography (SPECT) is a well-established and important in vivo technique to evaluate fundamental biological processes and unravel the role of neurotransmitter receptors in various neuropsychiatric disorders. Specific ligands are available for PET/SPECT studies of dopamine, serotonin, and opiate receptors, but corresponding development of radiotracers for receptors of glutamate, the main excitatory neurotransmitter in mammalian brain, has lagged behind. This state of affairs has persisted despite the central importance of glutamate neurotransmission in brain physiology and in disorders such as stroke, epilepsy, schizophrenia, and neurodegenerative diseases. Recent years have seen extensive efforts to develop useful ligands for molecular imaging of subtypes of the ionotropic (*N*-methyl-*D*-aspartate (NMDA), kainate, and AMPA/quisqualate receptors) and metabotropic glutamate receptors (types I, II, and III mGluRs). We now review the state of development of radioligands for glutamate receptor imaging, placing main emphasis on the suitability of available ligands for reliable in vivo applications. We give a brief account of the radiosynthetic approach for selected molecules. In general, with the exception of ligands for the GluN2B subunit of NMDA receptors, there has been little success in developing radiotracers for imaging ionotropic glutamate receptors; failure of ligands for the PCP/MK801 binding site in vivo doubtless relates their dependence on the open, unblocked state of the ion channel. Many AMPA and kainite receptor ligands with good binding properties in vitro have failed to give measurable specific binding in the living brain. This may reflect the challenge of developing brain-penetrating ligands for amino acid receptors, compounded by conformational differences in vivo. The situation is better with respect to mGluR imaging, particularly for the mGluR5 subtype. Several successful PET ligands serve for investigations of mGluRs in conditions such as schizophrenia, depression, substance abuse and aging. Considering the centrality and diversity of glutamatergic signaling in brain function, we have relatively few selective and sensitive tools for molecular imaging of ionotropic and metabotropic glutamate receptors. Further radiopharmaceutical research targeting specific subtypes and subunits of the glutamate receptors may yet open up new investigational vistas with broad applications in basic and clinical research.

## 1. Introduction

**Figure 1:**
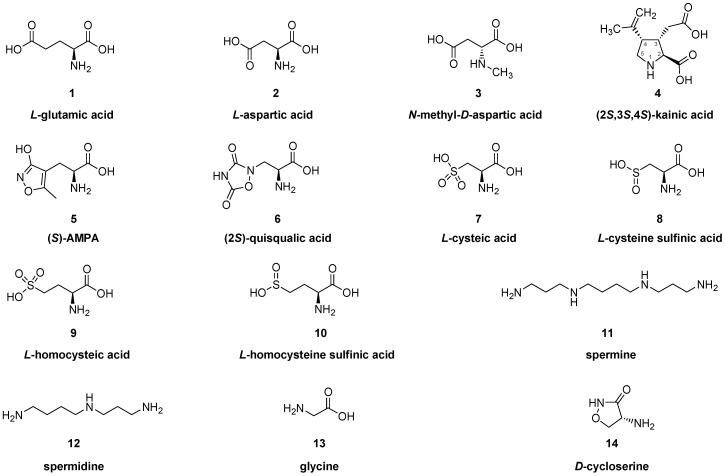
Chemical structures of excitatory amino acids (EAAs), sulfur-containing amino acids (SCAAs), and polyamines.

The first identification of the amino acid glutamate/glutamic acid (**1**) as a neurotransmitter was in the insect nervous system [1,2]. Glutamate occurs at a concentration of approximately 10 mM in mammalian brain [3], making it the most abundant amino acid, which reflects its presence both in metabolic and neurotransmitter pools [4]. Perhaps in keeping with its ancient evolutionary lineage as a neurotransmitter, glutamate possesses a wide variety of receptors, which have abundant expression on neurons and glia. The glutamate receptors fall into two broad categories, namely the ionotropic receptors, which are ligand-gated ion channels, and the metabotropic receptors (mGluRs), which have coupling to intracellular second messenger systems. The mGluRs have further subtypes, namely the excitatory type I receptors (mGluR1/mGluR5), the adenylate cyclase-inhibiting type II receptors (mGluR2, mGluR3), and the type III receptors (mGluR4, mGluR6, mGluR7, mGluR8). The ionotropic glutamate receptors, which are designated according to their excitatory amino acid ligands (Figure 1), i.e., *N*-methyl-*D*-aspartate (NMDA, **3**), kainate (**4**), and AMPA (**5**)/quisqualate (**6**) receptors, are ligand gated ion channels permissive to cation flux across the cell membrane. Given this enormously diverse pharmacology, no particular or singular action can be attributed to glutamate receptors the nervous system. In general, however, the ionotropic receptors mediate fast excitatory synaptic signaling and have a key role in synaptic plasticity, although their excessive activation provokes excitotoxicity, whereas the mGluRs are modulators/shapers of neuronal activity via their effects on intracellular cyclic adenosine monophosphate (cAMP) levels and other second messengers.

There are various reviews of the pharmacology of ionotropic [5,6] and metabotropic glutamate receptors [7,8]. There is also a burgeoning literature on the involvement of glutamate receptors in the pathophysiology and therapeutics of Alzheimer’s dementia [9], Parkinson’s disease [10], ischemic stroke [11], epilepsy [12], autoimmune diseases of the nervous system [13], and neuropsychiatric disorders such as schizophrenia [14,15,16], depression [17,18], and substance abuse/addiction [19]. As such, the glutamate receptors present a diverse range of important targets for molecular brain imaging by single photon emission computed tomography (SPECT) and positron emission tomography (PET). While there have been several reviews of this topic in recent years [20,21,22,23], we now present a systematic review of the state of development of glutamate receptor imaging, placing our emphasis on the findings of clinical studies, and on the lacunae remaining in the literature due to lack of specific tracers for many of these molecular targets. In selected cases, we give an account of the procedures for radiopharmaceutical synthesis.

### A Brief Note on the Various Endpoints and Units of Binding Studies

The comparison of findings with various PET and SPECT tracers in vivo requires some consideration of the various units and measures used to quantify radiotracer uptake and binding in the living brain. A neurochemist can measure the binding to membranes or brain sections of the radioligand over a range of concentrations in vitro. After subtracting the non-specific binding, the neurochemist can then calculate the saturation binding parameters, namely the B_max_, which is the absolute concentration of the binding sites in the sample (pmol g^−1^ or mols per liter of tissue), and K_D_, the affinity or half-saturating ligand concentration (mols per liter of solvent). Here, the experimentalist has full control of the free ligand concentrations and incubation conditions, which generally do not change during the binding experiment.

In PET and SPECT studies of the living organism, the radiotracer distributes throughout the various tissues of the body after its administration as an intravenous bolus injection. This immediately introduces a time-dependence of the concentration in blood, and further influences of hepatic metabolism and renal elimination on the radiotracer bioavailability. In the simplest form of quantitation, the PET or SPECT instrument reports the time–radioactivity curve in semiquantitative units of standard uptake value (SUV). This has units of percentage of the total injected dose per gram of brain tissue, sometimes with scaling to the corresponding uptake in a reference tissue devoid of specific binding (SUVR), if such a tissue exists for a given target. The SUV is a time-dependent parameter and is furthermore a composite index of specific and non-specific binding. Nonetheless, SUV serves as a convenient marker of how effectively the tracer can cross the blood–brain barrier (BBB). Successful SPECT or PET tracers usually attain an SUV of at least 1% of the total injected dose per gram of rodent brain and show higher SUV in brain regions enriched with the targeted binding site. Knowing the arterial input function by serial sampling with correction for radioactive metabolites, compartmental analysis of the dynamic brain curve gives estimates of microparameters in brain. Chief among these are the unidirectional blood–brain clearance of the radiotracer (K_1_), which has units of blood flow (ml g^−1^ min^−1^), the fractional rate constant for clearance of unbound tracer from brain (k_2_; min^−1^), and the association/dissociation rate constants with the cerebral target binding sites (k_3_/k_4_; min^−1^). Compartmental analysis also gives estimates of macroparameters, which are the composite of several microparameters, notably the net blood–brain clearance inclusive of irreversible trapping in brain (K_in_; mL g^−1^ min^−1^), which is defined as (K_1_*k_3_)/(k_2_ + k_3_). Calculated by linear graphical (Patlak-Gjedde) analysis of dynamic PET/SPECT data, K_in_ is quite distinct from the similarly named inhibition constant (K_i_; mols per liter) derived from a blocking study in vitro, which is more akin to the IC_50_, i.e., the plasma concentration of a competitor displacing 50% of the specific ligand binding in brain. Steady-state distribution volume ratios of a tracer concentration in brain to that in the blood (mL g^−1^) include the nonspecific binding (V_ND_), which is equal to the ratio K_1_/k_2_, and the total distribution volume (V_T_), which also includes the specific binding component, defined by the ratio k_3_/k_4_. The dimensionless binding potential (BP_ND_) represents specific binding as [(V_T_ − V_ND_)/V_ND_]. This returns us to the first principles of autoradiography, in that the BP_ND_ derived from PET measurements should be proportional to the ratio of the saturation binding parameters B_max_/K_D_ measured in vitro. If there is a brain region devoid of specific binding, this can serve for the reference tissue calculation of BP_ND_, thus avoiding the need for arterial sampling.

## 2. Glutamate Receptors

### 2.1. Ionotropic Glutamate Receptors (iGluR)

#### 2.1.1. Pharmacology of NMDA Receptor Ligands

**Figure 2:**
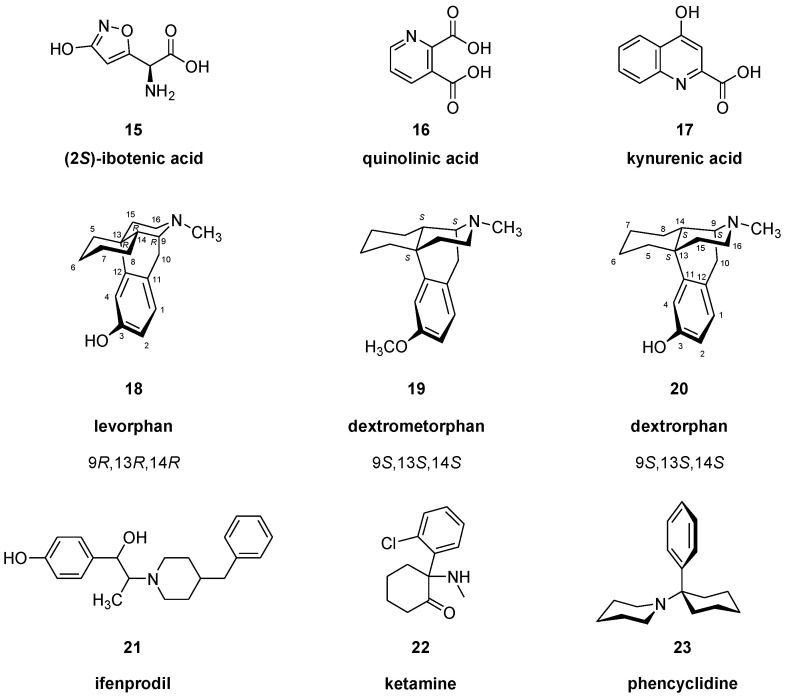
Chemical structures of some prototypic *N*-methyl-d-aspartate (NMDA) receptor ligands.

The first phase of molecular brain imaging dates to the late 1970s, with initial efforts to prepare radiopharmaceuticals labelled with iodine-123 for SPECT, or with short-lived positron-emitting radionuclides carbon-11 (t_1/2_ of 20 min) or fluorine-18 (t_1/2_ of 109 min) for PET brain imaging. The great preponderance of neurotransmitter molecular imaging studies have employed radioligands for dopamine, serotonin, and opioid binding sites, and progress in developing glutamate receptor ligands has picked up pace only in recent years. The first glutamate receptor studies targeted the NMDA receptor, which is a tetraheteromer consisting of two obligatory GluN1 and two GluN2 and/or GluN3 subunits, forming together a transmembrane pore for Na^+^/Ca^2+^ influx and K^+^ efflux. The receptor subunits are encoded by seven genes, with a single gene for GluN1, its transcript being subject to alternative splicing. Four GluN2 subunits (GluN2A, 2B, 2C, 2D) and two GluN3 subunits (GluN3A, 3B) encoded by different genes, respectively, further contribute to receptor diversity. There are many splice variants and several GluN2 transcripts, but the NMDA receptors are generally subject to allosteric modulation by Zn^2+^, the polyamines spermine (**11**) and spermidine (**12**), other proteins, and voltage dependent blockade of the cation channel by Mg^2+^ (for review, [24]). Activation of the NMDA receptor requires binding of the co-substrate amino acid *L*-glycine (**13**) to the GluN1 subunit and glutamate (**1**) (or NMDA, **3**) to the GluN2 subunit, at specific sites in the extracellular domain. In addition to NMDA (**3**) and glutamate (**1**), other agonists of NMDA receptors include *D*-cycloserine (**14**) (at the glycine-binding site), homocysteic acid (**9**), the *Amanita muscaria* toxin, ibotenic acid (**15**), and quinolinic acid (**16**). Among its antagonists are the endogenous tryptophan metabolite, kynurenic acid (**17**), the over-the-counter cough suppressant, dextromethorphan (**19**), and the antihypertensive agent ifenprodil (**21**). Figure 2 depicts some key structures.

##### Ligands for NMDA Receptor Ion Channels (PCP/MK801 Binding Site)

The NMDA receptor is a dimer of dimers, namely the GluN1 type paired with four known subtypes of GluN2 proteins, designated as A, B, C, and D. While the GluN1 confers the binding site for glycine/D-serine to the NMDA receptor complex, its association with the different glutamate-binding GluN2 sites results in ligand gated ion channels with distinct physiological properties. These properties differ with respect to Mg^2+^ block and cation conductance, as well as the kinetics of channel deactivation after prolonged agonism and concerning their intracellular trafficking pathways (for review see Willey et al. [25] and Vieira et al. [26]). The most abundant subunits in adult telencephalon are GluN2A and GluN2B, which seemingly act via distinct signaling pathways (Sun et al. [27]). There is relatively little knowledge about the functional properties of the GluN2C subunit, but its association with GluN1 results in an exclusively glycine-gated receptor. The GluN2D subunit is perhaps even more mysterious but may modulate cellular responses to ketamine that are relevant in the context of schizophrenia models ([28]). As shall be seen, the great preponderance of radioligands discussed in this section target the GluN2B subunit, thus leaving a vast territory of molecular imaging yet unexplored.

As a typical ligand gated ion channel, the NMDA receptor possesses an extracellular ligand-binding domain and an ion-conducting transmembrane domain, which is only open upon receptor activation. The NMDA receptor is pharmacologically complex, presenting at least four distinct binding sites, which are targetable by distinct classes of ligands. These sites include (A) the phencyclidine (PCP)/MK801 binding site in the transmembrane pore, (B) the glutamate binding sites on the extracellular side GluN2, (C) the co-agonist binding site for glycine (**13**) on the GluN1 subunit, and (D) allosteric modulator sites at the interface of the dimers comprising the complete tetramer. All of these sites present potential targets for PET/SPECT imaging. To this list might also be added the polyamine binding site, which modulates the binding of [^3^H]MK801 [29] and other intrachannel ligands.

While the synthetic opioid derivatives levorphan (**18**, Hoffmann-La-Roche, 1952), dextrometorphan (**19**, Hoffmann-La-Roche, 1954), and dextrorphan (**20**) share the same morphinan-skeleton, they differ significantly in their pharmacological profiles with respect to selectivity for the PCP/MK801 binding site in the NMDA channel. These compounds were synthesized from cyclohexanone in a ten-step procedure [30]. Levorphan (**18**), which has an *R*-absolute configuration at all of its asymmetric centres, is a potent opioid receptor agonist with an approximately seven-fold higher affinity than morphine [31]. Dextrometorphan (**19**), which is the (9*S*,13*S*,14*S*)-isomer, has no analgesic activity, but is commonly used as cough suppressant. Dextromethorphan (**19**) and its main metabolite dextrorphan (**20**) also show anticonvulsant and neuroprotective effects, which are apparently obtained by antagonism of NMDA glutamate receptors. Thus, stereochemistry of morphinan compounds determines their selectivity between opioid and NMDA receptors, and, as presented below, also with respect to sigma receptors.

The prototypic NMDA antagonist ligand [^3^H]MK801 binds to the inside of the channel with a K_D_ of 6 nM and B_max_ of 250 nM in rat brain membranes [32]. Its binding in cryostat sections from gerbil brain was most abundant in the hippocampus and cerebral cortex, and was substantially displaced by ketamine (**22**) and phencyclidine (**23**), but was unaffected by glutamate (**1**) or NMDA (**3**) [33]. The success of [^3^H]MK801 as a ligand in vitro led to the testing of [^18^F]fluoro-methyl-MK801 by PET (**25**), which showed slightly heterogeneous uptake in brain of living baboon [34]. However, its binding in vivo was not displaceable by challenge with phencyclidine (**23**) or excess non-radioactive MK801 (**24**), indicating a lack of specific binding.

Another widely used ligand for autoradiographic studies of NMDA receptors is the phencyclidine derivative [^3^H]-*N*-(1-[thienyl]cyclohexyl)3,4-piperidine ([^3^H]TCP), which binds to a single site in rat brain cryostat sections with an apparent affinity of 127 nM [35]. Its highest binding density was in the hippocampus, where it showed a complex laminar distribution; the specific binding of [^3^H]TCP (20 nM) was 320 nM in stratum radiatum membranes (assuming 10% protein). Neocortical binding was only about 160 nM, and striatal and thalamic binding was approximately 80 nM. Parallel studies with [^3^H]glutamate binding under supposedly NMDA-specific conditions (i.e., in the presence of quisqualic acid) showed an affinity of 160 nM, and a five-fold excess stoichiometry, which suggested that several glutamate molecules were required to fully activate the receptor. However, these studies were conducted in the absence of the co-ligand glycine, and so may not reflect the binding properties of glutamate in vivo.

**Figure 3:**
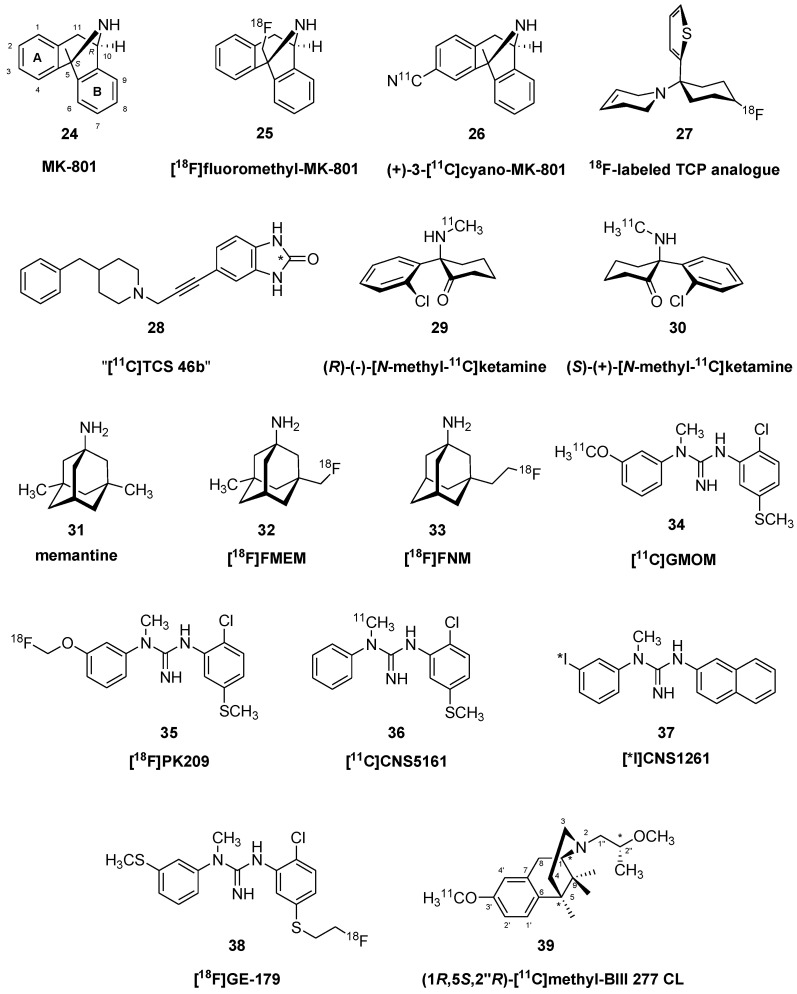
Chemical structures of labelled NMDA receptor ligands developed for the MK801/phencyclidine (PCP)-binding site of the NMDA ion channel.

Rodent studies with the phencyclidine derivative [^3^H]fluorothienyl-cyclohexylpiperidine indicated a small specific binding component in living brain, which was substantially increased by treatment with NMDA by intraperitoneal (i.p.) injection, suggesting a certain degree of activation-dependence of the binding [36]. Indeed, the normally low fraction of open channels in living brain is a key limitation of radioligands targeting the PCP/MK801 site. Around the same time, 1-[1-(2-thienyl)-4-([^18^F]fluoro)-cyclohexyl]-1,2,5,6-tetrahydropyridine (**27**) was prepared for imaging of the NMDA ion channel. Despite high affinity in vitro, there was only moderate uptake in brain of living rat (0.1% injected dose (ID) at 30 min post-injection [p.i.]), and little evidence of specific binding in brain of living rat or rhesus monkey [37], perhaps due to its dependence on activation of the NMDA receptors. Similarly, despite an IC_50_ value of 5 nM for blocking activation of the GluN1(A)/2B receptor subtype in vitro, 5-[3-(4-benzylpiperidin-1-yl)prop-1-ynyl]-1,3-dihydrobenzoimidazol-2-one (“[^11^C]TCS 46b”, **28**) labelled with carbon-11 on the benzoimidazolone ring had globally low uptake in brain of living rat, without any displacement by ifenprodil [38]. Some key structures of NMDA channel ligands are depicted in Figure 3.

Logan analysis of the uptake of [*N*-methyl-^11^C]ketamine enantiomers ((*R*)-(−)-**29**, (*S*)-(+)-**30**) in brain of living baboons showed a V_T_ of approximately 8 mL g^−1^ relative to the metabolite-corrected arterial input function. There was slightly preferential binding of the (*S*)-(+)-enantiomer (**30**), which was somewhat obscured by rapid washout of both tracers due to their rapid metabolism in vivo [39]. Others reported a transient stereoselective binding of [*N*-methyl-^11^C]ketamine ((*S*)-(+)-**30**) in brain of rhesus monkey, corresponding to an unusably low binding potential (BP_ND_) of 0.2–0.4 [40]. Nonetheless, by modifying the specific activity of their tracer, the authors were able to estimate a K_D_ of 7 µM in living brain, and a B_max_ of about 2 µM in living brain, which is about ten-fold higher abundance than NMDA receptor density in rat brain membranes. This discrepancy is doubtless due to various methodological factors, as well as physiological aspects of the state of binding sites occurring in the living organism.

In 2015, Salabert et al. published the radiosynthesis of the memantine (**31**) analogue [^18^F]fluoroethylnormemantine ([^18^F]FNM, **33**) and its biodistribution in rat brain [41]. The radiosynthesis of [^18^F]FNM (**33**) was achieved from 1-(*N*-Boc)-3-(2-tosyloxyethyl)-adamantane precursor in an automated radiochemical synthesis. The two-step procedure was realized using a Raytest SynChrom R & D fluorination module. In the first step of the radiosynthesis, the precursor, carrying a tosyloxy leaving-group was reacted in a nucleophilic substitution reaction with ^18^F^−^ (K_2_CO_3_, cryptand_222_, DMSO, 125 °C, 20 min). In the second step, the *N*-Boc protecting group was removed by hydrolysis with 6 N hydrochloric acid (110 °C, 10 min). The radiochemical yield was 10.5 ± 3% and the molar activity was > 355 GBq/μmol. [^18^F]FNM (**33**) had a K_i_ of 350 nM for the displacement of [^3^H]TCP from rat brain membranes, which was comparable to the values reported for memantine [42]. Uptake in brain of living rats was spatially uniform, with and DUV of 0.3% ID/g at 30 and 60 min post injection.

Others investigated the memantine derivative 1-amino-3-[^18^F]fluoromethyl-5-methyl-adamantane ([^18^F]-memantine, **32**), which had a high SUV in mouse brain (2.5% ID/g at 60 min), but was only 20% displaceable in vivo by pretreatment with MK801 [43]. Nonetheless, the authors proceeded to test their tracer in PET studies on healthy volunteers, finding a rather uniform binding throughout grey matter (V_T_ 15–20 mL g^−1^), which somewhat exceeded the accumulation in white matter [44]. They concluded that the tracer distribution in living human brain did not match the expected heterogeneous distribution of NMDA receptors.

A series of *N*,*N*′-diphenyl and *N*-naphthyl-*N*′-phenyl guanidine derivatives were prepared as potential PET ligands for the open state of the NMDA receptor, among which the 3-thiomethyl derivative had a K_i_ of 2 nM against [^3^H]MK801 binding (1 nM) in rat brain membranes [45]. Others tested a series of 80 *N*′-3-(trifluoromethyl)phenyl derivatives of *N*-aryl-*N*′-methylguanidines as displacers of [^3^H]MK801 binding, of which several were identified as having suitably high affinity [46]. Among these candidate tracers, [^11^C]*N*-(2-chloro-5-thiomethylphenyl)-*N*′-(3-methoxy-phenyl)-*N*′-methylguanidine ([^11^C]GMOM, **34**) showed a V_T_ in the range of 13–17 mL g^−1^ in baboon brain [47]. While this uptake in baboon brain was unaffected by MK801 treatment, there was some displacement in rat brain studies, and pretreatment with the glycine site ligand *D*-serine tended to increase the binding in vivo.

The fluorine-18 labelled analogue of GMOM, [^18^F]PK-209 (**35**) ([3-(2-chloro-5-(methylthio) phenyl)-1-(3-([^18^F]fluoromethoxy)phenyl)-1-methylguanidine] displaced [^3^H]MK801 from membranes with a K_i_ of about 20 nM, similar to that of [^11^C]GMOM (**34**) [48]. Preclinical PET studies in non-human primates showed extensive [^18^F]PK-209 (**35**) uptake in brain, slow washout, and rather rapid formation of radioactive plasma metabolites (only 20% parent at 20 min p.i.) [49]. The V_T_ was rather uniformly about 11 mL g^−1^ (including in the cerebellum), and pretreatment with MK-801 (**24**) (0.3 mg/kg) was without consistent effect on V_T_ in the three test monkeys. PET studies with [^18^F]PK-209 (**35**) in a group of ten healthy volunteers showed relatively high uptake, peaking at 20 min post injection, followed by washout with a half-life of about 90 min [50]. SUV images showed some spatial heterogeneity, with thalamus > striatum, cerebral cortex > cerebellum > white matter. The authors chose to model its uptake assuming irreversible binding relative to a metabolite-corrected arterial input, which yielded three parameters: the unidirectional blood–brain clearance K_1_ (0.45 mL g^−1^ min^−1^), non-specific distribution volume V_ND_ (7.8 mL g^−1^), and K_in_, the net blood-brain influx (0.016 mL g^−1^ min^−1^). There was no consistent effect of ketamine challenge on any of the endpoint parameters. Therefore, the authors were pessimistic about the reliability of the tracer for measuring NMDA receptor availability in vivo.

Tritiated (*N*-(2-chloro-5-methylthiophenyl)-*N*′-(3-methylthio-phenyl)-*N*′-methyl guanidine) (CNS-5161) bound to the NMDA receptor open channel with a K_D_ of 6 nM and a B_max_ of 330 nM (assuming 10% protein) in rat brain membranes; addition of glycine and glutamate to the medium increased the apparent affinity without altering the B_max_ [51]. Ex vivo studies showed time-dependent accumulation in rat forebrain regions, attaining a cortex/cerebellum ration of 1.4, which increased upon NMDA treatment and declined after MK-801 (**24**) treatment. Radiation dosimetry of [^11^C]CNS5161 (**36**) in humans was similar to that of widely used PET tracers, and a pilot investigation showed moderate uptake in human brain, peaking at 20 min post injection (effective dose equivalent: 0.0106 mSv/MBq (0.0392 REM/mCi)) [52]. The corresponding SPECT tracer *N*-(1-napthyl)-*N*′-(3-[^123^I]-iodophenyl)-*N*-methylguanidine ([^123^I]CNS-1261, **37**) showed high selectivity for NMDA receptors in rat brain, and its cerebral uptake was substantially higher on the side of a middle cerebral artery occlusion [53]. The authors suggested that this result indicated binding to the open state of NMDA receptors, which predominated in the infarcted and depolarized tissue on the side of the occlusion. [^123^I]CNS-1261 (**37**) uptake showed well-behaved kinetics in brain of healthy volunteers, attaining a V_T_ ranging from 9 mL g^−1^ in white matter to 16 mL g^−1^ in thalamus [54]. In a rare clinical investigation of NMDA receptors, [^123^I]CNS-1261 (**37**) was used to measure occupancy by ketamine in healthy individuals [55]. Clinical correlation analysis showed an association between the negative symptom scores of the brief psychiatric rating scale with higher occupancy by ketamine. In a study conducted in schizophrenia patients stably treated with clozapine, the V_T_ of [^123^I]CNS-1261 (**37**) was globally reduced by a third as compared to untreated patients and healthy controls [56]. This could indicate either simple competition of clozapine at NMDA sites, a treatment effect, or a disease-related down-regulation of binding. Using a binding index normalized to cortical tracer uptake, Pilowsky et al. found a significant reduction in NMDA binding in the left hippocampus in drug-free patients with schizophrenia, which was less prominent in patients taking clozapine or typical antipsychotics [57]. Furthermore, there was a significant inverse correlation between [^123^I]CNS-1261 binding and the severity of psychotic symptoms in those patients who were treated with typical antipsychotics. In drug-free patients, there was a significant positive correlation between tracer uptake in the middle inferior frontal cortex with illness duration. These results are generally in line with the NMDA hypofunction hypothesis of schizophrenia and suggest that antipsychotics may modulate the disease-related alterations.

[^18^F]GE-179 (**38**) is another bisarylguanidine NMDA receptor blocker, with structural resemblance to CNS5161 and GMOM. Preliminary blocking studies indicated a K_i_ of 2 nM for the displacement of [^3^H]tenocyclidine from the (phencyclidine, **23**) intrachannel binding site [58]. PET studies with [^18^F]GE-179 (**38**) in healthy humans showed good uptake and washout characteristics, and a V_T_ in the range of 8–14 mL g^−1^ in different brain regions of healthy human volunteers [59]. However, the authors were cautious about attributing its rather homogeneous distribution in brain to NMDA receptor binding. Indeed, rodent/non-human primate studies with displacement by MK-801 (**24**) and other drugs did not give compelling evidence for specific binding [60]. That discrepant finding provoked a lively discussion in the literature, with reference to the likely confounding effects of isoflurane + ketamine anesthesia, which is apt to reduce the availability of open channel binding sites in vivo [61]. With that in mind, others tested [^18^F]GE-179 (**38**) binding in living pig brain in the context of electrical stimulation of the hippocampus, while avoiding administration of ketamine as a potential competing anesthetic [62]. The stimulation apparently provoked an increase in the grey matter V_T_ from about 3.5 mL g^−1^ to 5 mL g^−1^, in the absence of any effect on perfusion as measured by [^15^O]-water PET in the same session. Autoradiographic studies ex vivo with [^18^F]GE-179 (**38**) showed persistent increases in binding in rat cerebral cortex after traumatic brain injury, which were interpreted to indicate activation of NMDA receptors in the injured tissue [63].

BIII 277 CL [(−)-(1*R*,5*S*,2″*R*)-3′-Hydroxy-*N*-(2″-methoxypropyl)-5,9,9-trimethyl-6,7-benzomorphan] with its 6,7-benzomorphan skeleton is a structural analogue of classical pharmacologically important compounds such as pentazocine, phenazocine, metazocine and *N*-allylnormetazocine (SKF10,047). The stereochemistry of receptor SKF10,047 isomers has a large effect on selectivity for different receptor types. Thus, the (−)-epimer prefers the μ- and κ-opioid receptors, the (+)-epimer binds to the sigma receptors and both epimers have some affinity for the NMDA receptors. Based on these findings, BIII 277 CL was developed at the pharmaceutical company Boehringer Ingelheim in the 1990s as a specific ion-channel blocker of the NMDA receptor-channel complex [64,65]. BIII 277 CL contains a phenolic hydroxyl group in position-3′and a 2″-*R*-methoxypropyl substituent on the nitrogen (*N*_2_).

In 2002, Kokic et al. described the preparation of [^11^C]methyl-BIII 277 CL (**39**) [66]. The radiosynthesis was accomplished by *O*-methylation of the 3′-*O*-desmethyl precursor (free base of BIII 277 CL) with [^11^C]iodomethane (K_2_CO_3_, DMSO, under nitrogen, 120 °C, 10 min). The tracer was prepared with a radiochemical yield of 15 ± 5% at the end of synthesis (EOS). The total synthesis took 45–50 min. The molar activity was 35–70 GBq/μmol and the chemical and radiochemical purity > 98%. Binding studies with [^11^C]methyl-BIII 277 CL (**39**) in rat brain membranes indicated K_D_ of 6 nM and B_max_ of 70 nM (assuming 10% protein), but very fast washout from brain of living pig, where the V_T_ was uniformly close to 1 mL g^−1^, indicating a lack of specific binding in vivo [66].

Based on the binding of bis(phenylalkyl)amines to the polyamine sites on NMDA subunits (more specifically the glycine-independent polyamine modulatory site), *N*-(3-(4-hydroxyphenyl) butyl-3-(4-[^11^C]methoxyphenyl)butylamine was tested as a potential PET tracer [67]. This tracer showed rather uniform binding in rat brain sections but was partially displaceable by spermine and by divalent cations in vitro.

##### Ligands for GluN2B Sites of the NMDA Receptor

**Figure 4:**
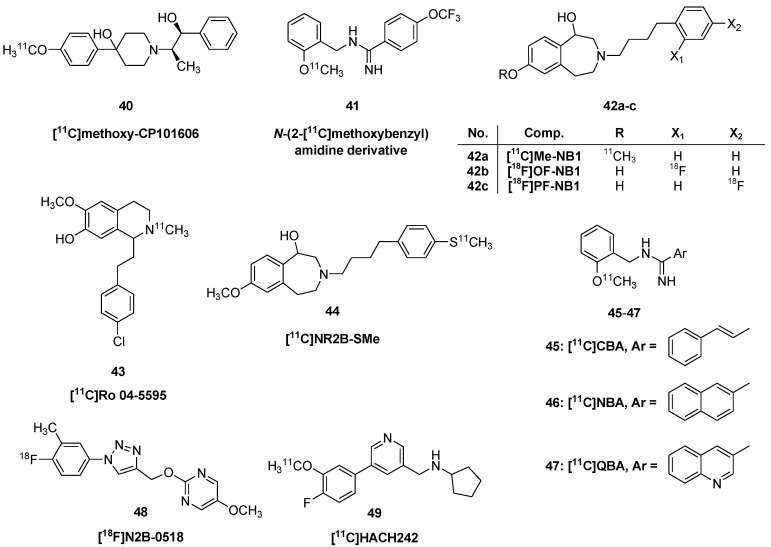
Structure of selected radioligands for the GluN2B subunit of NMDA receptors.

The general failure of earlier efforts to develop useful NMDA-channel binding PET tracers inspired a search for ligands targeting the GluN2B subunit of the NMDA receptor. Representative structures are shown in Figure 4. In one such study, the CP-101,606 analogue (+/−)threo-1-(4-hydroxyphenyl)-2-[4-hydroxy-4-(*p*-[^11^C]methoxyphenyl) piperidino]-1-propanol (**40**) was prepared [68]. The ligand had very high binding to the cortex, hippocampus and striatum in rat brain sections, but absent binding in the cerebellum, as expected for the GluN2B subtype [69]. However, PET examination in an awake rhesus monkey showed completely homogeneous uptake in living brain. Results of competition binding studies suggested that displacement by endogenous polyamines might interfere with its binding in vivo. The Glu2B-selective compound *N*-(2-[^11^C]-methoxy)benzyl-4-trifluoromethoxy-phenylamidine (**41**) showed forebrain-selective binding in rat brain in vitro, and moderate uptake in living brain (1% ID/g at 40 min), but the brain signal contained substantial amounts of labelled metabolites, which does not favor quantitation of specific binding [70].

In 2010, Wünsch and associates [71] reported the development of a new GluN2B-selective NMDAR antagonist, 7-methoxy-3-(4-phenylbutyl)-2,3,4,5-tetrahydro-1*H*-3-benzazepin-1-ol (WMS-1405), based on bond cleavage and reconstruction in the piperidine ring of ifenprodil (**21**). The preparation of WMS-1405 entailed a six-step reaction sequence starting from 3-methoxy-phenethylamine. The amine was reacted with four equivalents of tosyl chloride to yield *N*-(3-methoxyphenethyl)-4-toluenesulfonamide (pyridine, RT, 1.5 h, 97%), which was treated with ethyl bromoacetate (reflux, 20 h, 94%). Next, the resulting intermediate was converted to the corresponding free acid with sodium hydroxide (EtOH, H_2_O, reflux, 5 h, 91%). Cyclisation of the latter compound with P_2_O_5_ gave the desired 7-methoxy-3-benzazepine-1-one derivative as the main product. 9-Methoxy-3-benzazepine-1-one regioisomer and also 6-methoxy-*N*-tosyl-1,2,3,4-tetrahydroisoquinoline were isolated from the product mixture as minor by-products. The 7-methoxy-1*H*-3-benzazepine-1-one derivative was reduced with sodium borohydride to the corresponding 1*H*-3-benzazepine-1-ol compound. The latter compound was treated with Mg/MeOH to cleave the *N*-tosyl group and the formed secondary amine was *N*-alkylated to the desired WMS-1405 bearing a (CH_2_)_4_Ph substituent on the nitrogen.

In 2018, Krämer et al. [72] described the radiosynthesis and the evaluation of WMS-1405, which was codenamed [^11^C]Me-NB1 (**42a**), for the PET imaging of GluN1/GluN2B receptors. The radiosynthesis was preformed by selective alkylation of the desmethyl precursor NB1 [3-(4-phenylbutyl)-2,3,4,5-tetrahydro-1*H*-3-benzazepine-1,7-diol]. NB1 was prepared by 7-*O*-demethylation of WMS-1405 (Me-NB1) using boron tribromide in dichloromethane. NB1 was treated with [^11^C]iodomethane in the presence of Cs_2_CO_3_ as base in *N*,*N*-dimethylformamide (90 °C, 3 min). The molar activity was 290 ± 90 GBq/μmol at the end of synthesis and the radiochemical purity was > 99%. In PET imaging studies in rats, [^11^C]Me-NB1 exhibited a peak SUV value of four with a slightly faster washout from cerebellum than in forebrain structures. Blockade with various doses of eliprodil indicated a dose of 1.5 µg/kg at 50% of maximal occupancy (ID_50_), and a specific binding of about 50% of the total uptake. Various lines of evidence indicate insignificant interaction of the tracer with σ-receptors in vitro, whereas treatment with the σ_1_-receptor agonist (+)-pentazocine abolished the specific binding [^11^C]Me-NB1 (**42a**) in living rats, indicating an indirect effect of σ_1_-receptors on [^11^C]Me-NB1 binding. Very recently, Haider et al. [73] described the separation of the NB1 enantiomers by chiral high-performance liquid chromatography. Radiolabelling of the enantiopure precursors ((*R*)-NB1 and (*S*)-NB1) was achieved analogously to the method described earlier by the Ametamey-group for racemic [^11^C]Me-NB1 [72]. In addition to the radiochemical and radiopharmaceutical investigations, the absolute configuration of the reference standards (*R*)-Me-NB1 as well (*S*)-Me-NB1 was determined by X-ray crystallography and the stereostructure of the precursors was confirmed by circular dichroism spectroscopy. The in vivo results of the two enantiomers revealed for the *R*-enantiomer, (*R*)-[^11^C]Me-NB1, a high and a heterogenous accumulation in GluN2B-rich forebrain regions and dose-dependency of blockade by eliprodil. In contrast, the *S* enantiomer, (*S*)-[^11^C]Me-NB1, exhibited a homogenous distribution and no dose-response when eliprodil was applied as a GluN2B blocker.

Seeking to develop a fluorine-18 labelled analogue of [^11^C]Me-NB1 due to the inherently brief physical half-life of carbon-11, which limits its use only to centers with an on-site cyclotron, the Ametamey group evaluated a series of fluorinated analogues of Me-NB1 [74,75,76]. Two fluorine-18 labelled compounds, [^18^F]OF-NB1 (**42b**) and [^18^F]PF-NB1) (**42c**) (Figure 4) emerged as promising radioligands for imaging the GluN2B receptor. The binding affinity (K_D_) values for OF-NB1 and PF-NB1 towards the GluN2B subunits were 10.4 ± 4.7 nM and 10.4 ± 3.9 nM, respectively. Fluorine-18 labelling of both compounds was accomplished via copper-mediated radiofluorination in good radiochemical yields and molar activities ranging from 88–228 GBq/µmol using appropriate pinacol boronic ester precursors. Both radioligands displayed a heterogeneous and specific binding in GluN2B subunit-rich brain regions such as the cortex, striatum, hypothalamus and hippocampus in autoradiography experiments. PET imaging studies in Wistar rats showed a similar heterogeneous uptake. For both radioligands, a dose-dependent blocking effect was observed with CP-101,606 and resulted in an ID_50_ of 8.1 µmol/kg for [^18^F]OF-NB1 (**42b**) and 31 µmol/kg for [^18^F]PF-NB1 (**42c**).

Ro 04-5595 is a close structural analogue of the non-narcotic analgesic Versidyne [77] (Methofoline, Ro 4-1778/1). Both compounds are tetrahydroisoquinoline derivatives first synthesized in the early 1960s at Hoffmann-La-Roche. Versidyne, which has the same analgesic efficacy as codeine, had an indication for treatment of postoperative pain, but was withdrawn from the pharmaceutical market due to its opthalmic side effects. Ro 04-5595 contains a free phenolic hydroxyl group in position-7 instead of methoxy (Versidyne). In 2019, Jakobsson et al. [78] reported the synthesis of a new subtype selective GluN2B NMDA radiotracer, 1-(4-chlorophenethyl)-7-hydroxy-6-methoxy-2-[^11^C]methyl-1,2,3,4-tetra hydroisoquinoline ([^11^C]Ro 04-5595, **43**). The precursor for the radiosynthesis was *N*-desmethyl-Ro-04-5595 (1-(4-chlorophenethyl)-7-hydroxy-6-methoxy-1,2,3,4-tetrahydro isoquinoline). *N*-Methylation was performed with [^11^C]iodomethane in DMF (40 °C, 1 min) by application of the captive solvent method of Wilson et al. [79]. ([^11^C]Ro 04-5595, **43**) was synthesized with a radiochemical yield of 13 ± 3%, a radiochemical purity of 99%, and a molar activity of 12–43 MBq/nmol. The new tracer (**43**) showed a K_i_ of 2 nM relative to [^3^H]ifenprodil at GluN2B receptors in rat brain slices [78]. In brain of living rats, the tracer rapidly obtained a peak SUV of about 0.7, followed by rapid washout (t_1/2_ 20 min), which did not indicate much in the way of specific binding in vivo.

Enantiomers of (**44**) [S-Methyl-^11^C](±)-7-methoxy-3-(4-(4-(methylthio)phenyl)butyl)-2,3,4,5-tetrahydro-benzo[d]azepin-1-ol ([^11^C]GluN2B-SMe) were prepared and tested as GluN2Bsubunit ligands [80]. The compound exhibited 2 nM K_D_ for GluN2B receptors expressed in mouse fibroblasts. Of special interest was their demonstration that the ligand did not interact with σ_1_ receptors, which has been a limitation of several GluN2B ligands, including a series of tetra-hydro-1*H*-3-benzazepines [81] and fluorinated benzo[7]annulen-7-amines [82]. No radiolabelled metabolites of [^11^C]GluN2B-SMe (**44**) were detected in rat brain at 30 min after tracer injection, and preblocking with ifenprodil decreased the cerebral SUV from 3 to about 0.5. Of the two enantiomers, (*S*)-[^11^C]NR2B-SMe (**44**) seemed to have a somewhat superior binding signal in brain.

The benzamidazole 2-{[4-(4-iodobenzyl)piperidin-1-yl]methyl}benzimidazol-5-ol showed high affinity for the GluN2B subunit, with a K_i_ of 7 nM against [^3^H]ifenprodil binding to rat cortical synaptic membranes [83]. When labelled with iodine-125 it showed excellent properties for autoradiography in vitro, but only moderate cerebral uptake in rats (0.5% ID/g at 30 min post injection). Its binding in vivo was about 50% displaceable by treatment with the potent NR2B antagonist Ro 256,981.

The *N*-benzyl amidine derivatives 2-[^11^C]methoxybenzyl) cinnamamidine ([^11^C]CBA, **45**), *N*-(2-[^11^C]methoxybenzyl)-2-naphthamidine ([^11^C]NBA, **46**), and *N*-(2-[^11^C]methoxybenzyl)quinoline-3-carboxamidine ([^11^C]QBA, **47**) were tested as PET radioligands for GluN2B binding sites [84]. The three compounds were strongly displaced from rat brain sections by addition of the GluN2B blocker CP-101,606, and by spermine and Zn^2+^, but were unaffected by σ-ligands. However, the three tracers showed only moderate uptake in living rodent brain, attaining an SUV of about 0.33 at ten minutes, but showing little sign of specific binding in vivo.

Others have prepared an ^18^F-labelled potent antagonist, 2-((1-(4-[^18^F]fluoro-3-methylphenyl)-1*H*-1,2,3-triazol-4-yl)methoxy)-5-methoxy-pyrimidine ([^18^F]N2B-0518, **48**) as a ligand for PET imaging of the GluN2B subunits [85]. Autoradiography with [^18^F]N2B-0518 (**48**) gave excellent delineation of GluN2B sites in rat brain, with highest binding in frontal cortex and hippocampus, intermediate binding in striatum and thalamus, and nearly absent specific binding in cerebellum. Autoradiography in non-human primate brain revealed a laminar distribution in cortex, with highest binding in the superficial and deep layers. Despite these promising results in vitro, biodistribution studies in mice showed very rapid washout from brain, and little evidence of specific binding.

*N*-((5-(4-fluoro-2-[^11^C]methoxyphenyl)pyridin-3-yl)methyl)cyclopentanamin ([^11^C]HACH242, **49**) had a K_i_ of 12 nM relative to the displacement of [^3^H]ifenprodil from rat brain membranes [86]. Although its binding was only 30% displaceable by Ro25,6981 in mouse brain sections, binding measured ex vivo was consistently two-fold higher in the forebrain than in cerebellum, suggesting specific binding. PET imaging with [^11^C]HACH242 (**49**) was undertaken in a group of three nonhuman primates [87]. There was no consistent effect on the cerebral uptake upon treatment with radiprodil, which is a GluN2B negative allosteric modifier expected to reduce ligand binding to GluN2B sites. The authors did not attempt kinetic modelling but noted slower washout in frontal cortex (t_1/2_ circa 90 min) than in cerebellum (t_1/2_ circa 60 min), which they deemed consistent with a specific binding component in cortex.

##### Glycine Binding Site on the GluN1 Subunit of NMDA Receptors

**Figure 5:**
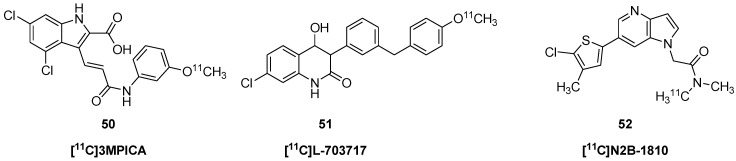
Structures of radiotracers for the glycine-binding site of the NMDA receptor.

A series of 2-carboxytetrahydroquinolines were screened as ligands for the glycine-binding site of the GluN1 subunit of the NMDA receptor [88] by measuring the displacement of [^3^H]-5,7-dichlorkynurenic acid from rat brain membranes. Others prepared [^11^C]-3-[2-[(3-methoxyphenylamino)carbonyl]ethenyl]-4,6-dichloroindole-2-carboxylic acid ([^11^C]3MPICA, **50**) as a potential PET radiotracer for the NMDA receptor glycine site [89]. See Figure 5 for structures.

The tracer had low uptake, attaining a rather uniform distribution of 0.15% ID/g at 60 min; the authors reported 25–50% displacement by pretreatment with non-radioactive 3MPICA, but attributed this to effects related to ligand binding at warfarin sites on serum albumin, rather than indication of specific binding. Intravenous co-injection of the 4-hydroxyquinolone tracer [^11^C]L-703,717 (**51**) along with warfarin increased the tracer uptake in mouse cerebellum, apparently due to competition against warfarin binding sites on plasma albumin [90]. Other mouse studies with [^11^C]L-703,717 (**51**)/warfarin co-injection showed selective labelling of the glycine binding site of a cerebellum-specific NMDAR ex vivo, but showed only telencephalic binding when measured in vitro, irrespective of the presence of the ε3 subunit responsible for the high cerebellar binding in vivo [91]. The discrepancy was attributed to regional differences in the brain concentration of *D*-serine, which is absent in cerebellum due to the high local activity of *D*-amino acid oxidase.

##### NMDA Allosteric Modulators

*N*,*N*-dimethyl-2-(1*H*-pyrrolo[3,2-b]″pyridin-1-yl)acetamide allosteric modulator ligands of the GluN2B subunit were prepared as potential PET tracers, among which [^11^C]N2B-1810 (**52**) showed “moderate” displaceable autoradiographic binding in rat telencephalon, and absent binding in cerebellum [92]. However, the tracer showed low (0.2% ID/g) and spatially uniform uptake in brain of living rats.

#### 2.1.2. AMPA Receptors

**Figure 6:**
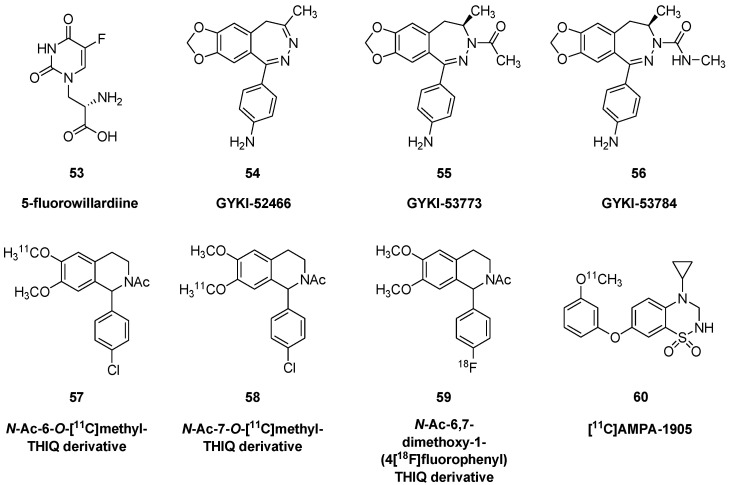
Structures of selected AMPA receptor ligands and non-competitive AMPA antagonists with 2,3-benzodiazepine scaffold.

Formerly known as quisqualate receptor, the AMPA receptor is so-named for its selective agonist, α-amino-3-hydroxy-5-methyl-4-isoxazolepropionic acid (**5**). For relevant structures, see Figure 6. The AMPA receptor is a ligand gated ion channel composed of four types of subunits, designated GluA1, 2, 3, and 4 (for review, [93]). Like the NMDA receptor, AMPA receptors are dimers of dimers, composed of a GluA2 dimer and one of the other dimers; changes in their composition, functional properties and trafficking across development critically determine synaptic plasticity [94]. Unlike NMDA receptors, each subunit of the AMPA receptor can bind its agonist, and pore opening requires binding to two or more subunits. The GluA2 subunit imparts to the channel a low permeability to Ca^2+^, so the open channel only supports Na^+^/K^+^ flux, and furthermore a voltage-dependent binding of polyamines to the GluA2 subunit modulates the K^+^ current. AMPA activation acts in concert with NMDA receptors to mediate long-term synaptic potentiation, whereby initial depolarization due to AMPA channel opening expels the Mg^2+^ that would otherwise block NMDA receptor-mediated currents. This allows an influx of Ca^2+^ that ultimately upregulates AMPA expression to increase the sensitivity of the membrane to glutamate.

5-Fluorowillardiine (**53**) is a natural product from the Willard acacia that acts as an excitotoxin in vivo. Rat brain membrane binding studies with (*S*)-[^3^H]-5-fluorowillardiine indicate a K_D_ of 7 nM (in the presence of KSCN) for AMPA receptors, and a B_max_ of about 120 nM (assuming 10% protein) [95]. Low concentrations of NBQX, domoic acid (generally considered a kainate ligand), AMPA, and glutamate displace its high affinity binding. Autoradiographic B_max_ of (*S*)-[^3^H]-5-fluorowillardiine ranged from 60 nM in thalamus to 440 nM in hippocampus. There was a 60% decline in (*S*)-[^3^H]-5-fluorowillardiine binding in cortex of rats with end stage hepatic failure, and parallel reductions in [^3^H]kainic acid binding sites, whereas NMDA binding sites labelled with [^3^H]MK-801 were unaffected [96]. Even if fluorowillardiine (**53**) and kainic acid (**4**) were available as PET tracers of high molar activity, their excitoxicity might preclude their use as imaging agents in vivo.

The first 5*H*-2,3-benzodiazepine derivative, (5-ethyl-1-(3,4-dimethoxyplenyl)-7,8-dimethoxy-4-methyl-5*H*-2,3-benzodiazepine) was prepared by Körösi and Láng [97] from 2-benzopyrylium salt via a monohydrazine-type intermediate. The 5*H*-2,3-benzodiazepine scaffold was formed in a base-catalyzed cyclocondensation. 1-(4-aminophenyl)-4-methyl-7,8-methylenedioxy-5*H*-2,3-benzodiazepine (GYKI-52466, **54**) [98,99,100], GYKI-53773 (**55**, talampanel) and GYKI-53784 (**56**) are structurally 2,3-benzodiazepine derivatives that represent selective non-competitive AMPA antagonists [101,102]. GYKI52466 (**54**), the first representative of this class of compounds, was first synthesized at the Institute for Drug Research (Budapest, Hungary, Gyógyszerkutató Intézet) and soon became the pharmacological standard of the field. The non-competitive mechanism of its action is important because these types of compounds act independently from glutamic acid (**1**), which is always present under endogenous conditions.

[^11^C]- and [^18^F]-labelled *N*-acetyl-1-aryl-6,7-dimethoxy-1,2,3,4-tetrahydroisoquinoline derivatives (**57**, **58**, **59**) were prepared as potential AMPA receptor ligands for PET imaging [103] based on findings reported by Gitto. However, there are no reports on binding properties in vivo for these compounds. Very recently, [^11^C]-labeled 4-cyclopropyl-7-(3-methoxyphenoxy)-3,4-dihydro-2*H*-benzo[*e*][1,2,4]thiadiazine 1,1-dioxide ([^11^C]AMPA-1905, **60**) was prepared as non-competitive AMPA antagonist ligand for PET imaging [104]. [^11^C]AMPA-1905 (60) was prepared from an *N*-Boc protected phenolic precursor, applying a one-pot two-step procedure. The precursor was reacted with [^11^C]iodomethane in *N*,*N*-dimethylformamide in the presence of tetrabutylammonium hydroxide (TBAOH) (80 °C, 5 min), followed by deprotection with 6 N HCl (80 °C, 5 min). The labelling was performed with a radiochemical yield of 22–28%, >99% radiochemical purity, and molar activity of 37 GBq/μmol. Its uptake in mouse brain was about 0.5% ID/g at 30 min post injection, with a rather uniform distribution to autoradiography ex vivo. Blocking studies with talampanel (55), an orally active AMPA antagonist, resulted in globally increased cerebral uptake, a finding that often indicates confounding effects of ligand displacement from peripheral binding sites. Very recently, Miyazaki et al. [105] developed a novel radioligand: 2-(2,6-difluoro-4-(2-(*N*-[^11^C]methylsulfonamido)ethylthio)-phenoxy)acetamide ([^11^C]K-2) for the PET imaging of AMPA receptors in the living human brain. The new radiotracer was prepared from a secondary amine type precursor (PEPA, K-1) with [^11^C]iodomethane in DMF (0.5 M NaOH, 80 °C, 5 min) in a simple radiosynthesis in high yield. [^11^C]K-2 is a potential tool to examine the role of AMPA receptors in neuropsychiatric disorders.

#### 2.1.3. Kainate Receptors

The kainate receptors are ionotropic non-NMDA receptors, which are selective to activation by the algal excitotoxic compound kainic acid (**4**, Figure 1). Kainate (**4**) is the most toxic of the excitatory amino acids, with a rank order of potency upon injection to the hippocampus kainate > ibotenate > NMDA > dihydrokainate > *d*,*l*-homocysteate > *l*-cysteate > *l*-aspartate > *l*-glutamate [106]. As such, kainic acid is a useful tool for neurochemical lesion studies. Like the other ionotropic glutamate receptors, the kainate receptor is a homomeric or heteromeric tetramer assembled from five possible subunits [107]. The ion channel of the kainate receptor is permissive to Na^+^/K^+^ flux, but the receptor seems also to possess coupling with a G-protein cascade, which is an unusual property for ionotropic receptors. Native kainate receptors expressed on neurons mediate slow excitation, distinct from the rapid depolarizations mediated by NDMA and AMPA receptors [108]. This property may be critically involved in encoding of temporal information and modulation of local and network spike activity, under the influence of auxiliary proteins like neuropilin and tolloid-like protein 1 (Neto1).

Autoradiography with [^3^H]kainic acid showed abundant specific binding in rat brain, which peaked at postnatal day 20 and declined slightly in adulthood [109]. The binding site was 50% more abundant in striatum than in cortex or cerebellum. Mean receptor densities labelled with [^3^H]kainic acid in rat telencephalon membranes were about 200 nM (assuming 10% protein), which is roughly similar to the corresponding concentrations of NMDA receptors labelled with [^3^H]MK-801, and about two-fold higher than the [^3^H]AMPA binding [110]. Displacement studies with the excitotoxin *L*-beta-oxalyl-amino-alanine (*L*-BOAA) (which is responsible for neurolathyrism) showed regional differences in the displacement of [^3^H]kainic acid binding in hippocampus, with lowest affinity in regions with high affinity for the shellfish excitotoxin domoic acid [111]. These findings indicate the presence of pharmacological heterogeneity of kainate receptors, despite the rather uniform displacement of [^3^H]kainate by the natural agonist glutamate, which showed an in vitro K_i_ of 1 µM across all hippocampal regions. So far, there has been no success in developing kainate receptor ligands with suitable properties for molecular imaging in vivo.

### 2.2. Metabotropic Glutamate Receptors (mGluRs)

The metabotropic glutamate receptors (mGluRs) are not ligand-gated ion channels but are typical members of the class C family of G-protein coupled receptors (GPCRs). The class C receptors consist of an N-terminal signal sequence that guides insertion into the plasma membrane, a hydrophilic extracellular agonist-binding domain that contains several cysteine residues, the seven transmembrane domains, and a C-terminal sequence dangling in the cytoplasm. Glutamate binding at mGluRs initiates dissociation of the intracellular heterotrimeric complex of GTP-binding protein subunits, which ultimately alters various metabolic processes in the post-synapatic neuron. There are eight known mGluR genes, which fall into three categories based on their sequence homology, i.e., the predominantly post-synaptic Group I (mGluR 1 and 5), the pre-and post-synaptic Group II (mGluR 2 and 3), and the predominantly presynaptic Group III (mGluR 4, 6, 7, and 8) [7]. The mGluRs are functional dimers in which the two “Venus flytrap” *N*-terminal domains have an open, inactive state, which converts to a closed state upon binding glutamate or other agonists; thus, the mGluRs have three functional states: open-open, open-closed, and closed-closed. The agonist-induced closure propagates a conformational change through the cytsteine-rich domain to the heptahelical transmembrane domain. Here, the transmembrane loops impart specificity for G-protein coupling, and present binding sites for allosteric modulators and phosphorylation sites for G-protein receptor kinases. Ultimately, agonist-induced conformational change propagates to the intracellular side, coupling to (inter alia) G_q_/G_11_ to activate phospholipase C_β_ and various downstream pathways (Group I mGluRs) or Gi/o proteins to inhibit adenylate cyclase or regulate ion channels (Group II and III mGluRs). The story of mGluRs is complicated further by possibilities for alternative splicing, and for protein–protein interactions through the C-terminal sequences, notably with Homer proteins of the post-synaptic density. The C-terminus of mGluR3 interacts with calmodulin and presents a phosphorylation site for protein kinase A [112].

The mGluRs interact importantly with ionotropic glutamate receptors. Notably, activation of protein kinase C by (Group I) mGluR5 participates in the long-term potentiation of ion currents mediated by NMDA receptors in the synapses between mossy fibers and CA3 pyramidal neurons of the hippocampus [113]. NMDA receptors in cerebral cortex undergo an activity-dependent developmental shift in their subunit composition from GluN2B in the first postnatal week of rats to predominantly GluN2A, which have faster kinetics; this shift requires co-activation of the mGluR5 and NMDA receptors [114]. The mGluR3 antagonist β-NAAG blocked the establishment of long-term depression in the dentate gyrus, whereas treatment with the putative endogenous agonist NAAG down-regulated long-term potentiation [115]. Treatment of rats with the (Group II) mGluR2/3 agonist LY379268 rescued the disruption of NMDA receptors induced by treatment with MK-801, and enhanced NMDA-provoked inward currents in rat brain slices [116]. The two receptors did not co-precipitate, but mGlu2/3R-activation of the Akt/GSK-3β pathway mediated the observed effects on NMDA receptors. On the other hand, treatment with LY379268 did not rectify working memory impairments in rats treated with MK801 (**24**) [117].

The functional association of mGluRs with NMDA receptors may present them as potential targets for the treatment of schizophrenia. Indeed, there have been tests of various mGluR ligands and allosteric modulators in animal models of schizophrenia and some such compounds have entered clinical trials [16]. Various lines of evidence imply that mGluR5 may also present a therapeutic target in Alzheimer’s disease [118]. In particular, mGluR5 seems to be a receptor of the neurotoxic amyloid-β42 peptide, and genetic deletion of mGluR5 interfered with the progression of amyloid deposition and mTOR phosphorylation and rescued cognitive impairment in a mouse model of Alzheimer’s disease [119]. The mGluRs are also potential therapeutic targets in diverse other conditions, including Parkinson’s disease [120], Huntington’s disease [121], fragile-X syndrome [122], seizure disorders [123,124], and substance abuse [125].

#### 2.2.1. Group I (mGluR1) Metabotropic Glutamate Receptors

**Figure 7:**
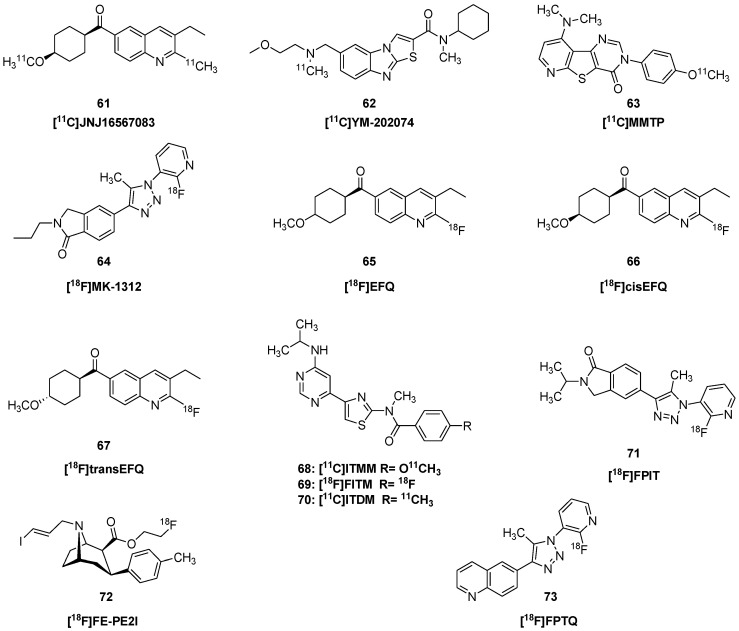
Structures of selected metabotropic glutamate receptor radiotracers (Group I, mGluR1).

(3-Ethyl-2-[^11^C]methyl-6-quinolinyl)(*cis*-4-methoxycyclohexyl)methanone, known as [^11^C]JNJ16567083 (**61**, Figure 7), shows high affinity for mGlu1 receptor (K_i_ = 0.87 nM) in rats and much lower affinity for the mGlu5 receptor (K_i_ = 2.4 µM). The radiotracer containing a ^11^CH_3_ group in position-2 of the quinoline scaffold was prepared from the corresponding 2-trimethylstannyl precursor. The reaction of the precursor with [^11^C]iodomethane under catalytic conditions (Pd_2_(dba)_3_, P(*o*-tolyl)_3_, DMF, 120 °C, 5 min) resulted in the radioligand with a radiochemical yield of 47 ± 17% and molar activity of 22.4 ± 8.4 GBq/μmol (607 ± 228 Ci/mmol). Dynamic rat PET studies with **61** revealed persistently two-fold higher uptake in the cerebellum than in forebrain regions, but this heterogeneity was blocked by treatment with an mGluR1 antagonist, whereas the mGluR5 antagonist was without any such effect [126].

*N*-cyclohexyl-6-{[*N*-(2-methoxyethyl)-*N*-methylamino]methyl}-*N*-methylthiazolo[3,2-a]-benzimidazole-2-carboxamide (YM-202074) had selectivity and high affinity for mGluR1 (K_i_ 5 nM), and autoradiography with [^11^C]YM-202074 (**62**) showed ten-fold higher binding in the rat cerebellum than in pons [127]. However, HPLC analysis of rat brain extracts showed considerable quantities of labelled radiometabolites at 30 min post injection. While pretreatment with JNJ16259685 globally displaced the specific binding, the uptake was relatively low, and the metabolite profile was not encouraging for its further use.

[*O*-methyl-^11^C]dimethylamino-3-(4-methoxyphenyl)-3*H*-pyrido[3′,2′:4,5]thieno[3,2-d]pyrimidin-4-one ([^11^C]MMTP, **63**) showed a K_i_ of 8 nM for mGluR1 in vitro [128], and was tested further using autoradiography in human brain sections, where it showed high specific binding in cerebellum [129]. PET studies in monkey brain showed high uptake in cerebellum, attaining 2% ID/g within minutes of injection, followed by rapid washout; the cerebellar binding was two-fold higher than in telencephalon.

[^18^F]MK-1312 was synthesized by the reaction of the chloro precursor with [^18^F]KF using microwave heating. Saturation binding studies with [^18^F]MK-1312 (**64**) indicated a K_D_ of 0.5 nM and a B_max_ of 53 nM in rhesus monkey cerebellum membranes, versus a K_D_ of 1 nM and B_max_ of 82 nM in human cerebellum [130]. PET recordings with [^18^F]MK-1312 (**64**) in rhesus monkey showed peak SUV of 2.5% ID/g at 20 min, followed by washout; two-tissue compartment kinetic analysis indicated rapid unidirectional clearance to cerebellum (K_1_; 0.33 mL g^−1^ min^−1^) and moderately fast association kinetics to its binding sites in living brain (k_3_; 0.03 min^−1^). Self-blocking studies indicated 50% occupancy at brain mGluR1 with a plasma concentration of 76 nM.

The non-competitive mGluR1 antagonist [^18^F]EFQ (**65**) (3-ethyl-2-[^18^F]fluoroquinolin-6-yl-(4-methoxycyclohexyl)methanone) exhibits a K_i_ of 2 nM for receptors in rat brain membranes, but only 25 nM K_i_ for cloned human receptors [131]. The tracer showed displaceable binding in rat cerebellum ex vivo, but rather homogeneous uptake (V_T_ 2.5–3.7 mL g^−1^) in a PET examination of baboon brain. This might have been due to rapid metabolism in baboon, where only 10% untransformed tracer remained in plasma at 15 min post injection, or possibly reflected insufficient binding affinity in humans and non-human primates. The two enantiomers of [^18^F]EFQ (**65**) were prepared and tested in mice and rats [132]. At ten minutes after injection of [^18^F]cisEFQ (**66**), the brain concentration was 3% ID/g in cerebellum and 2% ID/g in telencephalon, whereas [^18^F]transEFQ (**67**) showed relatively little uptake. The cerebral uptake of [^18^F]cisEFQ (**66**) was blocked by pretreatment with cisEFQ or JNJ16259685, but was unaffected by pretreatment with the mGluR5 ligand ABP688. Ex vivo autoradiography suggested relatively high binding in the thalamus. The most lipophilic of its main radiometabolites in mouse plasma constituted up to 20% of the brain activity, which is unfavorable for absolute quantitation of the PET signal.

*N*-[4-[6-(isopropylamino)pyrimidin-4-yl]-1,3-thiazol-2-yl]-4-[^11^C]methoxy-*N*-methyl-benzamide ([^11^C]ITMM, **68**), which was derived from the a potent negative allosteric modulator of mGluR1, has a K_D_ of 13.6 nM in vitro [133]. Analysis of the uptake of high molar activity [^11^C]ITMM (**68**) in rats with 90 min PET recordings gave V_T_ ranging from 2 mL g^−1^ in pons to 8 mL g^−1^ in striatum and 15 mL g^−1^ in cerebellum, calculated relative to a metabolite-corrected input function [134]. In a preclinical stroke study, declining [^11^C]ITMM (**68**) binding was indicative of the extent and progression of the ischemic brain injury [135].

The mGluR1 ligand 6-[1-(2-[^18^F]fluoro-3-pyridyl)-5-methyl-1*H*-1,2,3-triazol-4-yl]quinolone ([^18^F]FPTQ, **73**) was prepared for PET studies [136], having shown high selectivity and affinity for human (IC_50_ = 3.6 nM) and murine (IC_50_ = 1.4 nM) mGluR1 in vitro. [^18^F]FPTQ (**73**) showed good binding properties in vitro, with 20-fold higher binding in mouse cerebellum than in pons, and no displacement by mGluR5 antagonists. PET examination showed rapid influx into cerebellum, attaining 2.5% ID/g within minutes, followed by a rapid washout (t_1/2_ 20 min), with complete blockade upon treatment with MPEP (**74**). Less than 10% of plasma radioactivity was unmetabolized tracer at 30 min post injection, when only 67% of cerebellar activity was unmetabolized parent. A structurally similar compound bearing a triazole moiety, 1-(2-[^18^F]fluoro-3-pyridyl)-4-(2-isopropyl-1-oxo-isoindoline-5-yl)-5-methyl-1*H*-1,2,3-triazole ([^18^F]FPIT, **71**) showed binding in rat and monkey brain sections at a concentration of only 8 pM, although the saturation binding parameters were not presented [137]. The tracer showed slower brain kinetics in living rats, with a BP_ND_ of two in cerebellum calculated relative to cerebral cortex. However, the authors did not report on the metabolic stability of [^18^F]FPIT (**71**) in plasma, or the presence of labelled metabolites in the brain.

PET studies with 4-[^18^F]fluoro-*N*-[4-[6-(isopropylamino)pyrimidin-4-yl]-1,3-thiazol-2-yl]-*N*-methylbenzamide ([^18^F]FITM, **69**) showed progressive accumulation of radioactivity to at least 60 min post injection in telencephalic structures, when there was a 15:1 ratio of cerebellum relative to pons activity [138]. At 60 min post injection, 25% percent of plasma radioactivity remained as the parent tracer, and only traces of labelled metabolites were present in mouse brain, all of which favors quantitation of the PET signal. Dynamic PET recordings with [^18^F]FITM (**69**) showed faster kinetics in monkey brain, where peak radioactivity concentration (3.5% ID g^−1^) occurred at 30 min post injection. The ratio of cerebellum to pons activity was about 4:1 in monkey, but there was complete displacement of specific binding by pretreatment with JNJ16259685. Compartmental analysis indicated a V_T_ ranging from 2.4 in pons to 11.5 mL g^−1^ in cerebellum. The microparameters from a two-tissue compartment model indicated good blood–brain clearance (K_1_; 0.14 mL g^−1^ min^−1^) and rapid association to its binding sites in living brain (k_3_; 0.13 min^−1^).

In the first clinical application of [^11^C]ITMM PET (**68**), mGluR1 availability was compared in a patient with type VI spinocerebellar ataxia versus a group of healthy controls [139]. Relative to the pons, which is said to be devoid of mGluR1, the BP_ND_ of [^11^C]ITMM (**68**) in cerebellum ranged from two in the flocculus to six in the vermis of the healthy controls, and was reduced by 50% in the patient, reflecting the degeneration of Purkinje cells. A subsequent comparison of [^11^C]ITMM (**68**) and conventional [^18^F]FDG in a group of 12 patients with cerebellar ataxias gave similar sensitivities for disease detection, but the mGluR1 tracer BP_ND_ correlated better with clinical scores, suggesting a better depiction of the pathology [140]. A [^11^C]ITMM (**68**) PET study in a group of ten Alzheimer’s disease patients and age-matched healthy elderly controls did not indicate any AD-related difference in BP_ND_ anywhere in brain, nor did the binding in patients correlate with mini-mental state examination (MMSE) scores [141]. A comparison of 15 young (aged 26 years) and 24 elderly (aged 70 years) healthy controls did not reveal any group differences in [^11^C]ITMM (**68**) BP_ND_, nor were there any differences between men and women of either age group [142].

A comparison of *N*-[4-[6-(isopropylamino)-pyrimidin-4-yl]-1,3-thiazol-2-yl]-*N*-methyl-4-[^11^C]-methylbenzamide ([^11^C]ITDM, **70**) with [^11^C]ITMM (**68**) in nonhuman primates showed generally higher cerebral uptake for the former compound [143]. Both compounds had favorable metabolite profiles, with 60% parent fraction remaining at 15 min. However, the relative V_T_s between regions scarcely differed between the two tracers. Reference tissue quantitation obviously presents logistic advantages for any PET tracer, but its valid use depends on having a reference region nearly devoid of specific binding. Blocking studies with YM-202074 showed dose-dependent displacement of [^11^C]ITDM (**70**) in brain of living mice, including −36% in pons [144]. Indeed, Lassen graphical analysis relative to V_T_ gave a V_ND_ of 1.4 mL g^−1^, thus indicating a BP_ND_ of about five in cerebellum and calling into question the validity of pons as a non-binding reference region. That study was notable for comparing the non-invasive (image derived) input function with population-based metabolite correction, and a serial arterial sampling approach, which showed nearly identical V_T_ results.

A [^11^C]ITDM (**70**) study in mice in which V_T_ was calculated relative to an image-derived input function showed an approximately 20% decline across various brain regions in aged mice (16 months) compared to younger mice (6 months) [145], which stands in contrast to the [^11^C]ITMM (**68**) findings across human aging, cited above. In that same rodent study, examination of the Q175DN mouse model of Huntington’s disease showed persistently 17% higher [^11^C]ITDM (**70**) V_T_ compared to healthy wild type mice, indicating high levels of mGluR1 availability during the course of disease progression. In the A53T transgenic synclein model of Parkinson’s disease, deficits in motor behavior first manifest at 6–8 months of age, coincident with the start of nigrostriatal degeneration [146]. In a multitracer PET study of these A53T mice, there was over-expression of striatal mGluR1 at four months of age as measured by [^11^C]ITMM (**68**) PET, followed by a rapid decline at eight months, and nearly complete disappearance in elderly mice compared to non-carrier controls. Strikingly, the motor deficits in the transgenic mice correlated better with the loss of mGluR1 binding sites than with nigrostriatal degeneration measured with the dopamine transporter ligand [^18^F]FE-PE2I (**72**). The transgenic mice showed no abnormality in striatal binding of the selective mGluR5 [^11^C]ABP688 (**82**), which is discussed in detail in the following section. In a longitudinal [^11^C]ITDM (**70**) PET study of rats with pilocarpine-induced seizures, there was an acute 33% decline in the anterior part of the cerebellum, and a 20% decline after three weeks that was confined to the thalamus [147]. The mGluR1 findings were thus fairly restricted as compared to microglia PET examinations in the same mice with a TSPO ligand, which was nearly doubled at one and three weeks after the seizures.

#### 2.2.2. Group I (mGluR5) Metabotropic Glutamate Receptors

**Figure 8:**
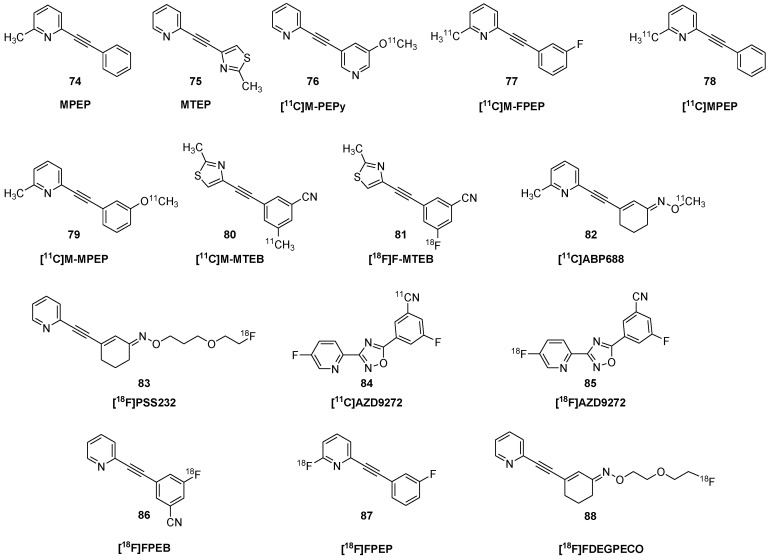
Structures of radiotracers for metabotropic glutamate receptors (Group I, mGluR5).

Early efforts towards developing mGluR5 PET ligands focused on diaryl alkynes, such as 2-methyl-6-(phenyl-ethynyl) pyridine (MPEP, **74**) and 3-[(2-methyl-1,3-thiazol-4-yl)ethynyl] pyridine (MTEP, **75**). Fore structures, see Figure 8. Autoradiographic studies with 3-[^3^H]methoxy-5-(pyridin-2-ylethynyl) pyridine ([^3^H]MPEPy) showed a K_D_ of 4 nM for binding to rat cortical membranes; the B_max_ ranged from 21 nM in hypothalamus to 83 nM in striatum, and specific binding was nearly absent in the rat cerebellum [148]. However, PET studies with 3-[^11^C]methoxyPEPy (**76**) showed little cerebral uptake in anesthetized baboons or rats [149]. Having excluded effects of anesthesia, the authors speculated that failure of the tracer in vivo was due to P-glycoprotein-mediated efflux. Ametamey et al. described the synthesis and biodistribution of 2-[^11^C]methyl-6-(3′-fluoro-phenylethynyl)-pyridine ([^11^C]M-FPEP, **77**), which obtained a rather homogeneous distribution in living rat brain [150]. [^11^C]MPEP (**78**) showed slightly higher uptake in rat cerebral cortex than in cerebellum at 60 min post-injection, and six-fold higher uptake in olfactory bulb, which was partially blocked by pretreatment with MPEP (**74**) (10 mg/kg) [151]; they found globally lower uptake of the methoxyphenyl derivative [^11^C]M-MPEP (**79**) and with [^11^C]methoxyPyEP (**76**). Blocking tended to increase globally the cerebral uptake of these tracers, suggesting displacement of the ligands from peripheral binding sites in rat. M-MTEB and F-MTEB showed sub-nM K_i_ against mGluR5 binding sites in vitro. The corresponding PET tracers [^11^C]M-MTEB (**80**) and [^18^F]F-MTEB (**81**) had rapid uptake in brain of living monkey, attaining SUV values as high as three, which was 75% displaced by pretreatment with MTEP (**75**) (1 mg/kg). However, displacement and autoradiographic saturation binding studies indicated an abundant binding density in rhesus cerebellum (B_max_ 24 nM), albeit less than in striatum (63 nM). This apparent species difference indicates that reference tissue quantitation is unsuitable for PET studies of mGluR5 in primate brain.

Based on these findings, Ametamey et al. developed (3-(6-methyl-pyridin-2-ylethynyl)-cyclohex-2-enone-*O*-[^11^C]-methyl-oxime) ([^11^C]ABP688, **82**), as a noncompetitive and highly selective antagonist molecular imaging of mGluR5 [152]. Saturation binding studies indicated a K_D_ of 2 nM and B_max_ of 23 nM (assuming 10% protein) in rat brain membranes. Initial small animal PET studies showed nearly four-fold higher uptake in rat hippocampus and striatum of wild type mice compared with mGluR5 knockout mice, thus confirming its specificity in vivo. [^11^C]ABP688 (**82**) has found application in numerous preclinical studies. PET and β-microprobe studies showed an effect of increasing mass on the apparent BP_ND_ in brain of isoflurane-anesthetized rats [153].

**Figure 9:**
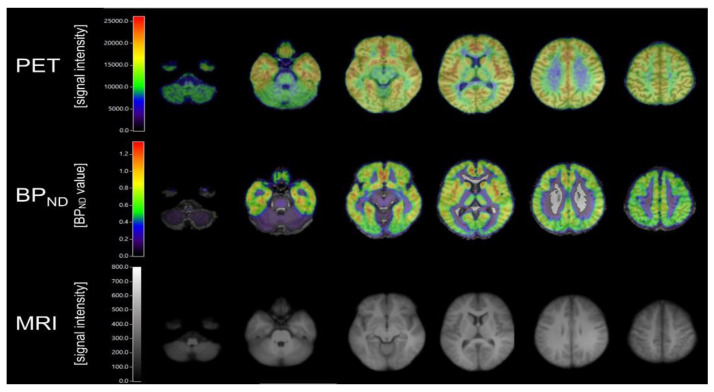
Representative mean images of [^11^C]ABP688 PET and corresponding MRI in a group of 23 healthy human subjects (Courtesy of the Neuroscience Research Institute, Gachon University, Incheon, South Korea).

However, pharmacological challenge with MK801 (**24**) or *N*-acetylcysteine, both of which treatments increase glutamate release, were without discernible effect on the binding in vivo. This result was in contrast to earlier findings of 10–20% reductions in [^11^C]ABP688 (**82**) BP_ND_ in rat brain following *N*-acetylcysteine challenge [154]. Other studies in isofluorane-anesthetized rats showed no effect on cerebral binding after challenge with ketamine (30 mg/kg) [155], despite results of human studies reported below that ketamine-challenge modulated [^11^C]ABP688 (**82**) via altered glutamate release. Similarly, rat PET studies with [^18^F]PSS232 (**83**), an [^18^F]-labelled analogue of ABP688, failed to show any effect of challenge with ketamine or ceftriaxone in vivo, nor was there any evidence for direct competition in brain slices [156]. On the other hand, treatment of anesthetized baboons with the glutamate releaser *N*-acetylcysteine decreased the magnitude of [^11^C]ABP688 (**82**) V_T_ by 20% in three of four animals, but only at the higher of two doses (100 vs. 50 mg/kg) [157]. Heterozygous knockout of the glutamate-synthesizing enzyme glutaminase has increased extracellular glutamate levels, which might be expected to reduce binding of [^11^C]ABP688 (**82**) to the allosteric site. However, glutaminase knockout mice showed unchanged mGluR5 availability in vivo, but decreased protein levels compared to Western blot analysis [158]. This was interpreted to be indicative of masking of the reduced mGluR5 expression by stimulated [^11^C]ABP688 (**82**) binding due to increased endogenous glutamate levels. A study of rats treated with ceftriaxone, an activator of the GLT-1 transporter that decreases extracellular glutamate levels, showed the expected increase in cerebral [^11^C]ABP688 (**82**) binding (+40%), consistent with an allosteric modulation of the PET tracer binding by glutamate [159]. Conversely, reductions of [^11^C]ABP688 (**82**) V_T_ in brain of living baboons could be used to detect the occupancy by different doses of intravenous fenobam [160]. Representative images of [^11^C]ABP688 PET in healthy humans are presented in Figure 9.

An alternate mGluR5 tracer of atypical structure, [^11^C]AZD9272 (**84**) had globally higher specific binding in brain of non-human primates as compared to [^11^C]ABP688 (**82**) [161]. Examined in detail by autoradiography with tritiated compounds, AZD9272 has distinctly higher binding in the ventral striatum, the substantial nigra, and some thalamic nuclei. Displacement studies showed complete displacement of [^11^C]AZD9272 (**84**) in vivo by fenobam, but only partial displacement by ligands of the MPEP (**74**) structure. Subsequent work showed it to have off-target binding to monoamine oxidase B, a property, shared with fenobam [161].

A study of AβPP transgenic mice showed no particular association between mGluR5 availability in vivo with Alzheimer’s disease model pathology, although post-mortem immunoblotting showed increases in mGluR5 protein levels that had been invisible to PET [162]. The 5xFAD mice, which bear five mutations linked to human Alzheimer’s disease, showed a 35% decrease in [^18^F]FPEB (**86**) BP_ND_ in hippocampus, cortex, and striatum, but this reduction only presented at the age of nine months, when severe amyloid pathology and behavioral deficits had already developed [163,164]. A longitudinal PET study in Huntington’s disease model mice showed a significant 14% reduction in striatum [^18^F]FPEB BP_ND_, and a somewhat lesser reduction throughout cerebral cortex compared to WT mice that was constant at three, six, and nine months [165]. The mGluR immunoreactivity was 37% lower in striatum and 17% lower in cortex of the transgenic mice, which might suggest some change in compartmental distribution of the receptor, such that the reductions measured in vivo were of lesser magnitude. In the pilocarpine model of seizure disorder there were 20–30% reductions in [^11^C]ABP688 (**82**) binding in striatum acutely after status epilepticus that had normalized in the chronic phase [166]; focal reductions persisted in the hippocampus and amygdala in the chronic phase, perhaps indicating a loss of neurons. Interestingly, an [^18^F]FPEB (**86**) PET study in transgenic mice with a mutation of superoxide dismutase occurring in human cases of amyotrophic lateral sclerosis (ALS) showed trebling of the BP_ND_ in striatum, hippocampus and doubling in cortex with progression to a neurologically advanced disease stage [167]. In the same mice there was a doubling of the specific binding of the TSPO ligand [^11^C]PBR28, a marker of activated microglia, thus linking the neurodegeneration of the ALS model with neuroinflammation and increased mGluR5 availability. Furthermore, PET studies with [^18^F]PSS232 (**83**) showed 20% increases in the distribution volume ratio (DVR) in various brain regions of mouse brain the day after challenge with lipopolysaccharide, which had completely normalized at five days in CD1 mice (which lack certain components of innate immune responses), while persisting in C57BL/6 mice [168]. In the same study, the authors reported three-fold high binding of [^18^F]PSS232 (**83**) in cryostat sections from cortex and basal ganglia samples from patients dying with ALS compared with control tissues.

PET studies in sapap3 knockout mice, which show progressive exaggeration of grooming behavior that models obsessive compulsive disorder, showed widespread 20% decreases in [^11^C]ABP688 (**82**) binding in association with their worsening behavior at nine months of age [169]. In this case, the decline in binding seen with small animal PET was not captured by semiquantitative autoradiography with [^3^H]ABP688 in vitro or by mGuR5 immunohistochemisty. An [^18^F]FPEB (**86**) PET study in Shank3B knockout mice, which present a behavior phenotype linked to autism spectrum disorder, showed 25% higher BP_ND_ compared to wild type mice in striatum, hippocampus and amygdala [170]; Western blotting confirmed these increases in protein level. A pilot human PET study showed higher [^18^F]FPEB BP_ND_ in the postcentral gyrus and cerebellum of male subjects with autism [171].

A retrospective analysis of a large series of [^11^C]ABP688 (**82**) studies in human subjects showed that the product was not stereochemically pure, but consisted of about 8% (*Z*)-isomer [172], which tended to reduce the magnitude of the BP_ND_ estimate, as had been shown earlier in rat studies [173]. Presumably, this effect may have accounted for the relatively high 11–21% test-retest variability reported in healthy volunteers [174]. Others investigated the within-subject variability of mGluR5 availability in a comparative PET study with [^11^C]ABP688 (**82**) and [^18^F]FPEB (**86**). The test-retest reproducibility of [^11^C]ABP688 (**82**) was lower when scans were not repeated on the same day, as was likewise seen with [^18^F]FPEB (**86**) PET, using a correction for residual radioactivity from a scan earlier in the day [175]. These results indicated that mGluR5 availability is inherently variable in the course of a single day, as had been first described for the [^11^C]ABP688 (**82**) V_T_, which tended to increase by 30% relative to a baseline scan two hours previously [176]. Forced sleep deprivation only slightly (+2.5%) increased the group mean magnitude of [^11^C]ABP688 (**82**) DV_norm_, but the increase was more pronounced in the subgroup with low baseline mGluR5 availability [177]. There were very high correlations between mGluR5 availability and electroencephalographic slow wave (0.25–1.0 Hz) oscillation power during non-rapid-eye-movement sleep, both in the baseline and rebound after sleep-deprivation conditions.

Other studies from the same research group showed that the whole brain [^11^C]ABP688 (**82**) BP_ND_ was 17% higher in men than in women, irrespective of the menstrual phase or hormonal contraceptive use of the female subjects [178]. Another [^11^C]ABP688 (**82**) study in 18 men and 13 women did not show any significant gender difference, and only a hint of a decline in BP_ND_ as a function of age [179]. That study employed a partial volume correction, making the results robust to any age-related loss of cortical volume. The authors of that study found some evidence of systematic asymmetry, with very slightly higher BP_ND_ in left hemispheric structures of healthy controls.

The binding of [^11^C]ABP688 (**82**) (DVR) was 27% lower in bilateral hippocampus of a group of (*n* = 9) patients with Alzheimer’s disease, and by about 20% in amygdala of the same patients, relative to a significantly younger non-demented control group (69 vs. 77 years) [180]. In a [^18^F]FPEB (**86**) PET study using a constant infusion design, the BP_ND_ was reduced by 43% in hippocampus of (*n* = 16) Alzheimer’s disease patients, compared to healthy age-matched and amyloid-negative controls [181]. The patients had early disease, as indicated by the mean MMSE score of 25. In a study with [^18^F]FPEB (**86**), patients with Parkinson’s disease (PD) had 20% higher BP_ND_ in putamen, hippocampus, and amygdala compared to healthy controls [182]. In the patients, there was a significant negative correlation (r = −0.51) between mGluR5 availability and dopamine transporter density in putamen. The results in human Parkinson’s disease patients are in contrast to findings in rats with parkinsonism due to a selective (6-OHDA) dopamine lesion, which showed reduced [^18^F]FPEB (**86**) binding in motor cortex and dorsolateral striatum [183], and in contrast to the unaltered [^11^C]ABP688 (**82**) binding in transgenic Parkinson’s disease model mice, cited above.

The binding of [^11^C]ABP688 (**82**) was reduced by about 20% in the hippocampus and amygdala of patients with temporal lobe epilepsy; these reductions persisted after successful surgical resection for alleviation of seizures [184]. The authors argued that the asymmetry index with this tracer was greater than that for [^18^F]FDG PET, thus indicating a role for mGluR5 PET in epileptogenic lesion localization. Another [^11^C]ABP688 (**82**) PET study from the same group showed reduced BP_ND_ in association with focal cortical dysplasia. Immunohistochemical examination of the resected cortical tissue confirmed loss of the normal cortical architecture [185].

A human PET study in ten healthy, non-smoking volunteers showed a mean 21% reduction in [^11^C]ABP688 (**82**) binding upon challenge with ketamine (**22**) infusions amounting to 0.8 mg/kg, which sufficed to produce a dissociative mental state [186]. A subsequent study by the same group using [^18^F]FPEP (**87**) showed lesser effects of ketamine on the magnitude of binding (V_T_/f_p_), but similar effect size as seen with [^11^C]ABP688 (**82**) [187]. The binding of [^11^C]ABP688 (**82**) was globally about 20% lower in a group of major depression patients of mean age 35 years as compared with age-matched healthy controls (in whom males were under-represented) [188]. Intravenous ketamine challenge rapidly improved mood in the patients, but provoked a similar decline in [^11^C]ABP688 binding (−14%) as that seen in healthy controls. The ketamine-induced reductions in BP_ND_ had returned to baseline in follow-up scans the next day.

An [^11^C]ABP688 (**82**) study showed no binding differences between (*n* = 20) patients with late-life depression and (*n* = 22) elderly healthy controls [189]. The authors conceded that their use of reference tissue quantitation might have compromised the reliability of their results. A PET study with [^11^C]ABP688 (**82**) showed widespread clusters of reduced mGluR5 availability in patients with major depressive disorder [190]. In that study, lower subcortical binding correlated with worse scores in the Beck anxiety inventory. Furthermore, post-mortem western blot analysis of individuals dying with major depressive disorder showed reduced mGluR5 protein levels in the cortical region BA 10. Likewise, another [^11^C]ABP688 (**82**) PET study showed 20% lower BP_ND_ (relative to cerebellum) in prefrontal cortex and in various cortical voxel clusters of a group of (*n* = 16) never-medicated young patients with major depressive disorder [191]. In that study, seed-based fMRI connectivity studies of the same patients reduced negative connectivity between certain cortical areas compared to that in the control group. In an [^18^F]FPEB (**86**) study of groups of (*n* = 29) individuals, the subgroup of post-traumatic stress disorder (PTSD) sufferers with suicidal ideation showed 25% higher V_T_ in amygdala, hippocampus, and frontal cortical regions compared to the healthy control group [192]. However, mGluR5 availability in major depressive disorder patients did not differ from that in the healthy control group, irrespective of suicidal ideation.

In a group of (*n* = 15) medicated patients with schizophrenia there were no regional differences in [^11^C]ABP668 (**82**) relative to age and (oversampled) smoking-matched healthy controls; smokers in both groups showed similar 24% lower global availability [193]. There was no group difference in [^11^C]ABP668 (**82**) binding in a contrast of patients with obsessive compulsive disorder compared with healthy controls, although there were some correlations with Yale–Brown obsessive-compulsive scale (Y-BOCS) scores [194]. In a group of (*n* = 16) patients with PTSD, the [^18^F]FPEB (**86**) V_T_ was 20% higher in dorsolateral prefrontal cortex, orbitofrontal cortex, and ventral striatum than in healthy controls, and the increases correlated with avoidance subscores in the patients [195]. A post-mortem arm of that study indicated elevated expression brain of the synaptic scaffolding protein SHANK1 in brain from PTSD patients, consistent with a more robust engagement of the mGluR5 with signal transduction pathways and NMDA receptor coupling.

Current smoking was associated with 20% lower [^11^C]ABP688 (**82**) binding (DVR) throughout the brain [196]. Studies in non-smoking patients with alcohol dependence showed 25% higher DVR in various brain regions, most notably in the amygdala, which DVR correlated inversely with craving scores [197]. A PET study with [^11^C]ABP688 (**82**) in groups of (*n* = 9) cocaine-dependent subjects and healthy age matched volunteers showed 20–30% lower BP_ND_ in the extended striatum and in amygdala and insula [198], with more pronounced reductions in those with longer abstinence. Another [^11^C]ABP688 (**82**) study of 15 cocaine-addicted subjects and healthy volunteers, matched for gender and smoking status likewise showed widespread 20% reductions in BP_ND_ relative to the (imperfect) cerebellum reference region [199]. MR spectroscopy of the same subjects did not show any group difference in the Glx (glutamate + glutamine) peak but did show a significantly higher *N*-acetylaspartate peak in the cocaine-addicted subjects.

A PET study with [^11^C]ABP688 reported higher mGluR5 availability in temporal lobe regions in male patients with alcohol use disorder (mean alcohol abstinence period: 25 ± 18 days) than in controls [197]. A longitudinal PET study with [^1^^8^F]FPEB PET showed lower mGlu5 availability in the limbic regions, posterior cingulate cortex, caudate of recently abstinent alcohol-dependent subjects (mean alcohol abstinence period: 7.3 ± 4.0) than in controls; however, after six months of alcohol abstinence, subjects showed increased mGluR5 availability comparable to the levels observed in controls, suggesting a possible reversibility [200]. A very recent [^11^C]ABP688 PET study showed that mGluR5 availability is low in the striatum, orbitofrontal cortex, and insula in youth (age range: 18–20) at elevated risk for substance use disorders, particularly those who frequently used cannabis [201].

(*E*)-3-(pyridin-2-ylethynyl)cyclohex-2-enone-*O*-2-(2-[18]F-fluoroethoxy)ethyl-oxime, ([^18^F]-FDEGPECO, **88**) is a promising mGluR5 ligand of somewhat distinct structure compared the diaryl compounds discussed above. It showed good binding properties in rat brain slices and dose-dependent displacement in hippocampus and striatum by treatment with M-MPEP [202]. The closely related compound (*E*)-3-(pyridine-2-yletheynyl-1)-cyclohex-2-enon-*O*-(3-(2-[^18^F]-fluoroethoxy)propyl)-oxime ([^18^F]PSS232, **83**) had a K_D_ of 3 nM in vitro [203]. Ex vivo studies with [^18^F]PSS232 in rats showed rapid kinetics favorable for evaluation within 70-min PET recordings, despite the presence of some displaceable binding in the cerebellum reference region. The first [^18^F]PSS232 (**83**) study in humans showed well-behaved cerebral kinetics relative to the arterial input function, which was stably quantifiable with dynamic recordings lasting only 45 min [204]. The considerable metabolic stability of the tracer, which remained 60% intact in plasma samples collected at 90 min after injection, presents a distinct advantage for PET studies with this tracer.

#### 2.2.3. Group II (mGluR2 and mGluR3) Metabotropic Glutamate Receptors

**Figure 10:**
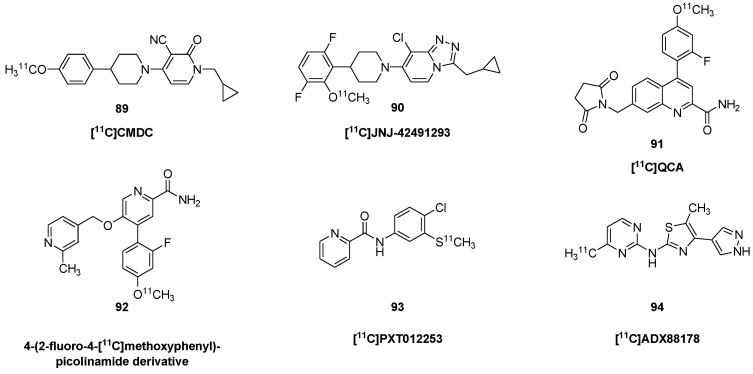
Structures of radiotracers for Group II (mGluR2 and mGluR3) and Group III (mGluR 4, 6, 7 and 8).

The first successful Group II ligand was the antagonist [^3^H]-2*S*-2-amino-2-(1*S*,2*S*-2-carboxycyclopropan-1-yl)-3-(xanth-9-yl) propionic acid ([^3^H]LY341495, which had a binding affinity (K_D_) of 1.7 nM for mGluR2 and a 0.75 nM for mGluR3 [205]. For structures, see Figure 10. Evaluations in rats showed only a hint of displaceable binding ex vivo, due to the tracer’s poor permeability to the BBB, which the authors attributed to hindering effects of the carboxylic acid moiety [206]. Ma et al. reported the radiosynthesis of 1-(cyclopropylmethyl)-4-(4-[^11^C]methoxyphenyl)-piperidin-1-yl-2-oxo-1,2-dihydropyridine-3-carbonitrile ([^11^C]CMDC, **89**) for the PET imaging of mGluR2 [207]. The compound is a methoxy-analogue of the compound JNJ-40068782, with a K_D_ value of 10 nM for recombinant human mGluR2 in vitro [208]. Autoradiographic studies with [^11^C]CMDC (**89**) in vitro showed abundant displaceable binding in rat brain sections. Dynamic PET acquisitions in rats showed an early peak uptake of about 0.5 SUV units, followed by slow washout. Displacement studies and examinations in transgenic knockout mice showed only traces of specific binding in brain. About 75% of the brain radioactivity was untransformed by parent compound at 30 min post injection, which may be too low for reliable quantitation of the PET data.

A series of 7-(phenylpiperidinyl)-1,2,4-triazolo[4,3-a]pyridines were also tested as positive allosteric modulator ligands of mGluR2 [209]. Among these, 8-chloro-3-(cyclopropylmethyl)-7-[4-(3,6-difluoro-2-methoxyphenyl)-1-piperidinyl]-1,2,4-triazolo[4,3-a]pyridine ([^11^C]JNJ-42491293, (**90**) showed promise in small animal PET studies. Further studies of **90** showed a K_D_ of 10 nM at human mGluR2 in vitro (23), and good binding properties in rat brain cryosections [210] and showed moderate cerebral uptake and self-displaceable binding in rat PET studies. However, there was no difference between uptake in wild type and mGluR2 knockout rats, indicating that the preponderance of cerebral binding was to some unidentified off-target site.

The negative allosteric modulator 7-((2,5-dioxopyrrolidin-1-yl)methyl)-4-(2-fluoro-4-[^11^C]methoxyphenyl)-quinoline-2-carboxamide ([^11^C]QCA, **91**) was prepared and tested as a tracer for imaging mGluR2 [211]. Using a functional assay involving calcium influx, QCA treated specifically modulate the effect of glutamate at mGluR2, exerting no such effect at mGluR3 [211]. In vitro autoradiographic studies showed high binding in cerebral cortex, hippocampus, striatum and cerebellum, but little displaceable binding in the midbrain and pons. Unfortunately, [^11^C]QCA (**91)** PET examination showed practically no influx into brain of living rats. Another mGluR2 negative allosteric modulator, 4-(2-fluoro-4-[^11^C]methoxyphenyl)-5-((2-methylpyridin-4-yl)methoxy) picolinamide (**92**), showed displaceable binding in rat brain sections [212]. PET examination showed it to have good initial uptake in rat brain, rapidly attaining a peak SUV of about 0.8, followed by rapid washout, but little sign of heterogenous or displaceable binding in vivo. Uptake was higher in mice lacking the P-glycoprotein, but still without significant specific binding.

The peptide *N*-acetlyaspartylglutamate (NAAG), which is among the most abundant neurotransmitters in the brain, acts with a low potency agonist at NMDA receptors, but more potently activates mGluR3 on post-synaptic neurons and astrocytes [213], although others have noted that contamination of NAAG samples by glutamate may lead to spurious findings [214]. The activation of mGluR3 generally inhibits the formation of cAMP, with net effects depending on its association with inhibitory or excitatory synapse. Activation of mGluR3 potentiates the effects of mGluR5 signaling on neuronal Ca^2+^ influx, thus indicating a functional partnership to the two subtypes [215]. In turn, the mGluR5 has functional linkages to NMDA receptors via intracellular scaffolding proteins, such as Homer, Shank, and postsynaptic density-95, which are key regulators of synaptic function [216]. The present lack of specific ligands for mGluR3 is unfortunate, given the reported and varying interactions between mGluR3, mGluR5 and NMDA receptors.

#### 2.2.4. Group III (mGluR4, mGluR6, mGluR7 and mGluR8) Metabotropic Glutamate Receptors

Immunohistochemical examination with specific antisera against mGluR4 showed a rank order of staining intensity cerebellar cortex > striatum = substantia nigra > cortex = thalamus [217]. Electron microscopic examination localized the receptor to presynpaptic boutons of type I and type II synapses. Activation of the mGluR4 inhibits the release of GABA and glutamate in parts of the basal ganglia, while tending to decrease excitatory transmission in cerebral cortex, thus drawing attention to it as a possible therapeutic target in Parkinson’s disease (see [218]).

The *N*-(methylthiophenyl)picolinamide mGluR4 positive allosteric modulator ligand [^11^C]PXT012253 (formerly [^11^C]KALB012, **93**) was characterized as having a K_i_ of 3 nM in a competition binding assay in vitro, and showed promising properties for brain imaging in rodent studies [219,220]. For structures, see Figure 10. The PET tracer also had rapid uptake in non-human primate brain, attaining a peak SUV of about five within five minutes after tracer administration, followed by rapid, but heterogeneous, washout [221]. Analysis by a one-tissue compartment model indicated V_T_ ranging from 4.4 mL g^−1^ in cerebellum to 6.3 mL g^−1^ in striatum, and 7.7 mL g^−1^ in thalamus. Brain penetration of the mGluR4 positive allosteric modulator PXT002331 was confirmed in occupancy studies using [^11^C]PXT012253 (**93**) in macaque monkeys [218]. Recently, the Brownell group developed a [^18^F]-labelled version, namely *N*-(4-chloro-3-(([^18^F]fluoromethyl-*d*_2_)thio)phenyl)picolinamide for imaging mGluR4 in the brain [220]. In that study, preliminary PET examination in rats showed good brain uptake and considerable spatial heterogeneity.

There are considerable discrepancies between the cerebral distribution of the picolinamide binding sites described above and the immunohistochemical localization of group III mGluRs, thus suggesting the occurrence of off-target binding of that class of ligands. With this disagreement in mind, others have tested the positive allosteric modulator ligand 5-methyl-*N*-(4-[^11^C]methyl pyrimidin-2-yl)-4-(1*H*-pyrazol-4-yl)thiazol-2-amine ([^11^C]ADX88178, **94**), based on findings that the non-radioactive compound potentiated the response of mGluR4 to glutamate with an EC_50_ of 4 nM [222]. Rat studies ex vivo showed adequate tracer uptake in brain, but little in the way of specific binding.

Activation of the mGluR6 inhibits forskolin-stimulated cAMP production, with *L*-2-amino-4-phosphonobutyrate (*L*-AP4) and *L*-serine-*O*-phosphate having ten-fold greater agonist potency than *L*-glutamate [223]. In situ hybridization indicated that mGluR6 expression is restricted to the retina. The mGluR7 receptor, which is also sensitive to *L*-AP4, showed moderate mRNA expression in neocortex, limbic cortex, hippocampus, and many other regions including septum, amygdala, specific hypothalamic nuclei and locus coeruleus [224]. The selective mGluR8 agonist (*S*)-3,4-dicarboxyphenylglycine has analgesic properties, perhaps in-keeping with the distribution across the pain neuraxis [225]. There are no reports of ligands or allosteric modulators suitable for imaging of mGluR6, 7, or 8.

## 3. Conclusions and Outlook

We have reviewed the present state of development of PET/SPECT probes for glutamate receptor imaging, including both ionotropic and metabotropic receptors, and discussed the suitability of the various tracers for reliable quantitation in the living brain. The diverse pharmacology of glutamate receptors, together with the central role of glutamatergic neurotransmission in brain function present a multitude of targets for molecular imaging. Quantitative imaging of ionotropic receptors is still difficult, despite early success with the intrachannel NMDA receptor SPECT ligand, [^123^I]CNS-1261 in clinical studies of schizophrenia. There has been some recent progress in developing suitable radioligands for PET imaging of the GluN2B subtype of the NMDA receptor. There has been some success in preclinical development of ligands for AMPA receptors in the living brain, but molecular imaging of kainite receptors remains unattainable; this seems remarkable, given their central role in synaptic plasticity. There has been rather better progress in mGluR imaging, particularly for the mGluR5 subtype, which has a functional link to NMDA receptors via intracellular scaffolding proteins. This has enabled rather extensive clinical investigations of mGluR5 availability in disorders such as drug abuse/addiction, depression, and PTSD. It is important to note that these disorders are heterogeneous and have high comorbidity with each other. For example, smoking history must be strictly controlled in PET studies with the most widely used mGluR5 ligand, [^11^C]ABP688, and there is some evidence of rapid diurnal changes in mGluR5 availability. However, several members of the mGluR family including mGluR3, mGluR6, mGluR7, and mGluR8 remain uninvestigated by molecular imaging. 

## Abbreviations: Acronym

**Compound**	**No.**	**Name: Synonyms**
[^11^C]ABP688	**82**	3-(6-methyl-pyridin-2-ylethynyl)-cyclohex-2-enone-*O*-[^11^C]methyl-oxime
[^11^C]ADX88178	**94**	5-methyl-*N*-(4-[^11^C]methylpyrimidin-2-yl)-4-(1*H*-pyrazol-4-yl) thiazol-2-amine
[^11^C]AMPA-1905	**60**	4-cyclopropyl-7-(3-[^11^C]methoxyphenoxy)-3,4-dihydro-2*H*-benzo[*e*][1,2,4] thiadiazine 1,1-dioxide
BIII 277 CL		(−)-(1*R*,5*S*,2″*R*)-3′-Hydroxy-*N*-(2″-methoxypropyl)-5,9,9-trimethyl-6,7-benzomorphan
B_max_		maximal binding capacity
Boc		*t*-Boc, *tert*-butoxycarbonyl group
BP_ND_		binding potential relative to the no displaceable compartment
Bq		Becquerel
cAMP		cyclic adenosine monophosphate
[^11^C]CBA	**45**	*N*-(2-[^11^C]methoxybenzyl) cinnamamidine
[^11^C]CMD	**89**	1-(cyclopropylmethyl)-4-(4-[^11^C]methoxyphenyl)-piperidin-1-yl-2-oxo- 1,2-dihydropyridine-3-carbonitrile
[*I]CNS1261	**37**	*N*-(1-napthyl)-*N*′-(3-[*I]-iodophenyl)-*N*-methylguanidine ([*I]CNS-1261
[^11^C]CNS-5161	**36**	*N*-(2-chloro-5-methylthiophenyl)-*N*′-(3-methylthio-phenyl)-*N*′-[^11^C]methylguanidine
Dextrometorphan	**19**	(+)-3-methoxy-*N*-methyl-morphinan, (9*S*,13*S*,14*S*)-dextrometorphan, CAS RN: [125-71-3]
Dextrorphan	**20**	(+)-3-hydroxy-*N*-methyl-morphinan, (9*S*,13*S*,14*S*)-dextrorphan, CAS RN: [125-73-5]
EEAs		excitatory amino acids
[^18^F]EFQ	**65**	3-ethyl-2-[^18^F]fluoroquinolin- 6-yl-(4-methoxycyclohexyl)methanone
[^18^F]FE-PE2I	**72**	(*E*)-*N*-(3-iodoprop-2-enyl)-2β-carbo-[^18^F]fluoroethoxy-3β-(*p*-tolyl)-nortropane
[^18^F]FDG		2-deoxy-2-[^18^F]fluoro-*D*-glucose, CAS RN: [86783-82-6]
[^18^F]FITM	**69**	4-[^18^F]fluoro-*N*-[4-[6-(isopropylamino)pyrimidin-4-yl]-1,3-thiazol-2-yl]- *N*-methylbenzamide
[^18^F]FMEM	**32**	1-amino-3-[^18^F]fluoromethyl-5-methyl-adamantane, [^18^F]-memantine,
[^18^F]FNM	**33**	[^18^F]fluoroethylnormemantine
EOS		end of synthesis
[^18^F]FPEB	**86**	3-[^18^F]fluoro-5-[(pyridin-3-yl)ethynyl]benzonitrile
[^18^F]FPEP	**87**	2-[^18^F]fluoro-6-[(3-fluorophenyl)ethynyl]pyridine
[^18^F]FPIT	**71**	1-(2-[^18^F] fluoro-3-pyridyl)-4-(2-isopropyl-1-oxo-isoindoline-5-yl)- 5-methyl- 1H-1,2,3-triazole
[^18^F]FPTQ	**73**	6-[1-(2-[^18^F]fluoro-3-pyridyl)-5-methyl-1*H*-1,2,3-triazol-4-yl]quinolone
[^11^C]GMOM	**34**	*N*-(2-chloro-5-thiomethylphenyl)-*N*′-(3-[^11^C]methoxy-phenyl)-*N*′-methylguanidine
GYKI-52466	**54**	1-(4-aminophenyl)-4-methyl-7,8-methylenedioxy-5*H*-2,3-benzodiazepine, CAS RN: [192065-56-8, as hydrochloride]
GYKI-53773	**55**	(*R*)-(−)-1-(4-aminophenyl)-3-acetyl-4-methyl-7,8-methylenedioxy-3,4,dihydro-5*H*- benzodiazepine, talampanel, LY-300164, CAS RN: [161832-65-1]
GYKI-53784	**56**	(*R*)-(−)-1-(4-aminophenyl)-3-methylcarbamoyl-4-methyl-7,8-methylenedioxy- 3,4,dihydro-5*H*-benzodiazepine, LY-303070, CAS RN: [161832-71-9]
[^11^C]HACH242	**49**	*N*-((5-(4-fluoro-2-[^11^C]methoxyphenyl)pyridin-3-yl)methyl)cyclopentanamine
IC_50_		inhibition constant required for displacement of 50% of radioligand
ID		injected dose
iGluR		ionotropic glutamate receptors
[^11^C]ITDM	**70**	*N*-[4-[6-(isopropylamino)-pyrimidin-4-yl]-1,3-thiazol-2-yl]-*N*-methyl- 4-[^11^C]-methylbenzamide
[^11^C]ITMM	**68**	*N*-[4-[6-(isopropylamino)pyrimidin-4-yl]-1,3-thiazol-2-yl]-4-[^11^C]methoxy- *N*-methyl-benzamide
[^11^C]JNJ16259685	**61**	(3-ethyl-2-[^11^C]methyl-6-quinolinyl)(cis- 4-methoxycyclohexyl)methanone
K_D_		dissociation constant or half-saturation concentration
K_i_		inhibition constant in vitro
[^11^C]L-703717	**51**	7-chloro-4-hydroxy-3-[3-(4-[^11^C]methoxybenzyl)phenyl-2-(1*H*)-quinolone
L-AP4		*L*-2-amino-4-phosphonobutyrate
Levorphan	**18**	levorphanol, (−)-3-hydroxy-*N*-methyl-morphinan, (9*R*,13*R*,14*R*)-levorphan, CAS RN: [77-07-6]
MDD		major depressive disorder
mGluR		metabotropic glutamate receptor
[^11^C]methyl-BIII 277 CL	**39**	(−)-(1*R*,5*S*,2″*R*)-3′-[^11^C]methoxy-*N*-(2″-methoxypropyl)-5,9,9-trimethyl-6,7-benzomorphan; [2*R*-[2α,3(*R*), 6α]-1,2,3,4,5,6-hexahydro-3-(2-methoxypropyl)-6,11,11-trimethyl-2,6-methano-9-[^11^C]methoxy-3-benzazocine
[^11^C]M-FPEP	**77**	2-[^11^C]methyl-6-(3′-fluoro-phenylethynyl)-pyridine
[^11^C]Me-NB1	**42a**	7-[^11^C]methoxy-3-(4-phenylbutyl)-2,3,4,5-tetrahydro-1*H*-3-benzazepine-1-ol
[^18^F]MK-1312	**64**	1-(2-[^18^F]fluoro-3-pyridyl)-4-(2-propyl-1-oxo-isoindoline-5-yl)-5-methyl-1*H*-1,2,3-triazole
[^11^C]M-MPEP	**79**	2-(2-(3-[^11^C]methoxyphenyl)ethynyl)pyridine
[^11^C]MMTP	**63**	dimethylamino-3(4-[^11^C]methoxyphenyl)-3*H*-pyrido[3′,2′:4,5]thieno- [3,2-d]pyrimidin-4-one
[^11^C]MPEP	**78**	2-[^11^C]methyl-6-(2-phenylethynyl)pyridine
[^11^C]3MPICA	**50**	3-[2-[(3-[^11^C]methoxyphenylamino)carbonyl]ethenyl]-4,6-dichloroindole-2-carboxylic acid
NAAG		*N*-acetylaspartylglutamic acid, CAS RN: [3106-85-2]
[^18^F]N2B-0518	**48**	2-((1-(4-[^18^F]fluoro-3-methylphenyl)-1*H*-1,2,3-triazol-4-yl)methoxy)-5-methoxypyrimidine
NB1		3-(4-phenylbutyl)-2,3,4,5-tetrahydro-1*H*-3-benzazepine-1,7-diol
[^11^C]NBA	**46**	*N*-(2-[^11^C]methoxybenzyl)-2-naphthamidine
NMDA		*N*-methyl-*D*-aspartic acid, CAS RN: [6384-92-5]
NMDAR		*N*-methyl-*D*-aspartate receptor
[^11^C]N2B-1810	**52**	2-(6-(5-chloro-4-methylthiophen-2-yl)-1*H*-pyrrolo[3,2-b]pyridin-1-yl)-*N*-(λ^1^-methyl-^11^C)-*N*-methyl-acetamide
[^11^C]NR2B-SMe	**44**	[S-methyl-^11^C](±)-7-methoxy-3-(4-(4-(methylthio)phenyl)butyl)-2,3,4,5-tetrahydro-1*H*-benzo[d]azepin-1-ol
[^18^F]OF-NB1	**42b**	3-(4-(2-[^18^F]fluoro-phenyl)butyl)-2,3,4,5-tetrahydro-1*H*-3-benzazepine-1,7-diol
[^18^F]PF-NB1	**42c**	3-(4-(4-[^18^F]phenyl)butyl)-2,3,4,5-tetrahydro-1*H*-3-benzazepine-1,7-diol
PCP	**23**	phencyclidine, CAS RN: [77-10-1]
[^18^F]FPDEGPECO	**88**	(*E*)-3-(pyridin-2-ylethynyl)cyclohex-2-enone *O*-2-(2-[18]F-fluoroethoxy)ethyl oxime
PET		positron emission tomography
[^18^F]PK-209	**35**	([3-(2-chloro-5-(methylthio)phenyl)-1-(3-([^18^F]fluoromethoxy)phenyl)- 1-methylguanidine]
[^18^F]PSS232	**83**	(*E*)-3-(pyridine-2-yl-etheynyl-1)-cyclohex-2-enon-*O*-(3-(2-[^18^F]-fluoroethoxy)propyl) oxime
PTSD		post-traumatic stress disorder
[^11^C]PTX012253	**93**	*N*-(4-chloro-3-([^11^C]methylthio)phenyl)picolinamide, [^11^C]KALB012
[^11^C]QBA	**47**	*N*-(2-[^11^C]methoxybenzyl)quinoline-3-carboxamidine
[^11^C]QCA	**91**	7-((2,5-dioxopyrrolidin-1-yl)methyl)-4-(2-fluoro- 4-[^11^C]methoxyphenyl) quinoline-2-carboxamide
[^11^C]Ro 04-5595	**43**	1-(2-(4-chlorophenyl)ethyl)-7-hydroxy-6-methoxy-2-[^11^C]methyl-1,2,3,4-tetrahydro- isoquinoline
SCAAs		sulfur-containing amino acids
SKF10,047		*N*-allyl-normetazocine; 2′-hydroxy-5,9-dimethyl-*N*^2^-allyl-6,7-benzomorphan; CAS: [825594-24-9]
SPECT		single photon emission computed tomography
SUV		standard uptake value
TBAOH		tetrabutylammonium hydroxide, CAS RN: [2052-49-5]
TCP		*N*-(1-[thienyl]cyclohexyl)3,4-piperidine, tenocyclidine, CAS RN: [2150098-1]
Versidyne		1-(2-(4-chlorophenyl)ethyl)-6,7-dimethoxy-2-methyl-1,2,3,4-tetrahydro-isoquinoline, methopholin, NIH-7672, CAS RN: [2154-02-1]
V_T_		total distribution volume
[^11^C]YM-202074	**62**	*N*-cyclohexyl-6-{[*N*-(2-methoxyethyl)-*N*-[^11^C]methylamino]methyl}-*N*-methyl- thiazolo[3,2-a]-benz imidazole-2-carboxamide
